# Multisensory Neuromorphic Devices: From Physics to Integration

**DOI:** 10.1007/s40820-025-01940-9

**Published:** 2026-01-12

**Authors:** An Gui, Haoran Mu, Rong Yang, Guangyu Zhang, Shenghuang Lin

**Affiliations:** 1 College of Semiconductors (College of Integrated Circuits), Hunan University, Changsha, 410000 People’s Republic of China; 2 Songshan Lake Materials Laboratory, Dongguan, 523808 People’s Republic of China; 3 School of Microelectronics Science and Technology, Sun Yat-sen University, 519082 Zhuhai, People’s Republic of China

**Keywords:** Neuromorphic computing, Multisensory signals, Physical mechanism, Multisensory fusion, Synapse

## Abstract

The increasing complexity of intelligent sensing environments, driven by the growth of Internet of Things technologies, has created a strong demand for neuromorphic systems capable of real-time, low-power multisensory perception. Traditional sensory architectures, constrained by single-modal processing and centralized computing, struggle to meet the requirements of diverse and dynamic input conditions. Multisensory neuromorphic devices offer a promising solution by mimicking the distributed, event-driven processing of biological systems. Recent efforts have explored synaptic devices and material systems that respond to various input modalities, including visual, tactile, thermal, and chemical stimuli. However, challenges remain in signal conversion, encoding compatibility, and the fusion of heterogeneous inputs without loss of unisensory information. This review provides a comprehensive overview of the physical mechanisms, device behaviors, and integration strategies that underpin signal processing in neuromorphic hardware. We highlight synaptic mechanisms conducive to cross-modal interaction, analyze representative signal fusion approaches at the device level, and discuss future directions for constructing efficient, scalable, and biologically inspired multisensory neuromorphic systems.

## Highlights

- This review provides a comprehensive overview of the physical mechanisms, device behaviors, and integration strategies that underpin multimodal signal processing in neuromorphic hardware.
- This review examines implementation strategies for multimodal integration, including signal fusion methods and processing techniques for handling cross-modal stimuli.
- This review categorizes multimodal neuromorphic devices into three distinct frameworks and comprehensively discusses their respective advantages and limitations.

## Graphical abstract

**Figure:**
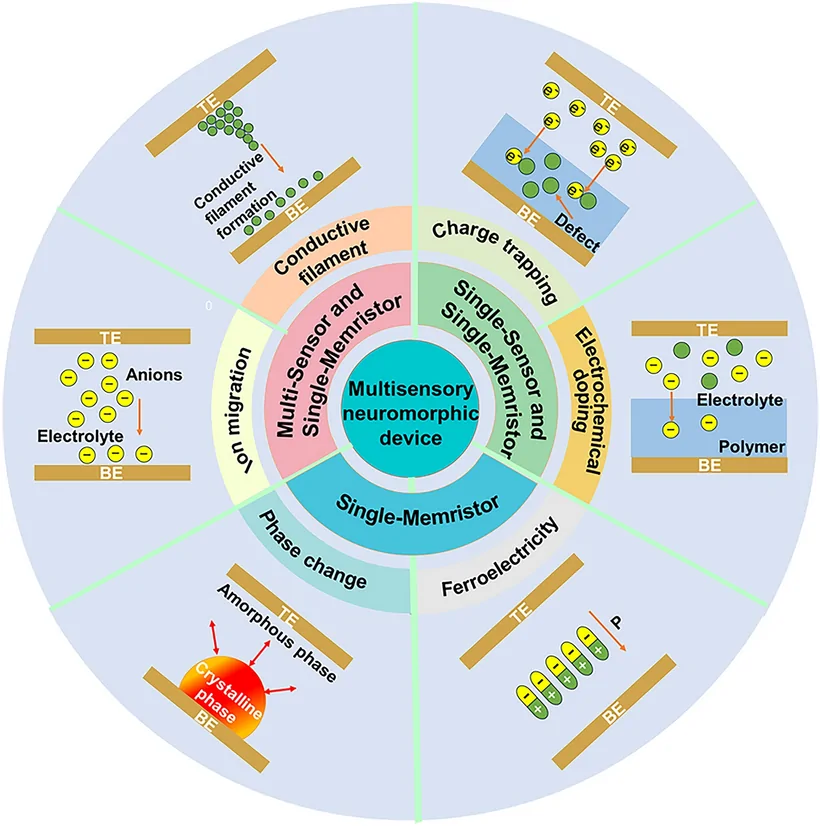

## Introduction

With the rapid development of IoT technologies, modern sensing systems are increasingly required to collect multimodal signals—such as visual, temperature, humidity, gas and pressure in real time and under dynamic, noisy environments [1, 2]. Traditional sensors, with their single-modal architectures and centralized data processing pipelines, often struggle to meet these demands due to limited signal compatibility, inherent stochasticity, and inadequate environmental adaptability [3–5]. To address these limitations, multisensory neuromorphic devices have attracted growing interest for their ability to emulate the brain’s parallel, distributed, and adaptive information processing capabilities [6–9]. By incorporating mechanisms such as synaptic plasticity and distributed computation, these devices perform direct, in-memory fusion of heterogeneous sensory inputs at the hardware level [10–12]. This biologically inspired approach enhances perception accuracy, reduces latency, and improves energy efficiency, which holds significant promise for real-time, energy-constrained applications such as autonomous vehicles, wearable electronics, and intelligent robotics [13–16].

Biological systems perform multisensory integration through highly interconnected and adaptive neural networks [17–21]. In these systems, synapses act as dynamic sensing and processing units, regulating signal transmission between neurons in response to various external stimuli [22, 23]. Changes in chemical flux within synapses modulate synaptic weights, enabling plasticity and adaptive learning based on multisensory input patterns [24, 25]. Sensory neurons and synapses integrate signals from different modalities into coherent spike trains, which propagate through the brain to support perception and decision-making [26]. This decentralized, parallel processing mechanism has inspired the development of artificial neuromorphic devices designed to emulate the functionalities of biological neurons and synapses [27, 28]. For example, neuromorphic systems incorporating heterogeneous sensory components, such as ferroelectric memristors and piezoresistive thin films, have been proposed. These systems achieve synchronous acquisition and spike-based encoding of multimodal inputs, thereby overcoming the serial bottlenecks of traditional architectures [29, 30]. These biologically inspired implementations lay the groundwork for more efficient and adaptive multisensory computing in artificial systems.

Recent studies have demonstrated neuromorphic devices capable of processing multimodal information by integrating visual, tactile, thermal, and chemical inputs into a unified hardware platform [31–33]. These systems mark a significant departure from traditional architectures by enabling in-memory and event-driven computation [28, 34, 35]. However, challenges remain in signal conversion and fusion. Diverse modalities differ in physical properties and encoding requirements, often requiring additional conversion modules that increase latency and energy consumption [36]. Moreover, naïve fusion strategies can result in the loss of key unisensory information, particularly under unbalanced input conditions [37]. Material incompatibility and limited integration scalability also hinder device performance and system robustness [38]. These limitations underscore the need to deepen our understanding of the physical mechanisms and fusion principles governing multisensory neuromorphic systems.

In this review, we provide an overview of multisensory neuromorphic devices. We analyze the operating principles by which different physical mechanisms respond to diverse input signals across visual, tactile, thermal, and chemical modalities. We then have discussed the requirements of mechanisms for achieving multimodal integration and which types of physical mechanisms are more conducive to multimodal fusion. Additionally, we examine implementation strategies for multimodal integration, including signal fusion methods and processing techniques for handling cross-modal stimuli. Finally, we highlight challenges in data conversion and fusion, and discuss future directions for constructing versatile neuromorphic systems with parallel processing capabilities.

## Mechanisms and Characteristics of Neuromorphic Devices

### Characteristics of Neuromorphic Devices

**Fig. 1:**
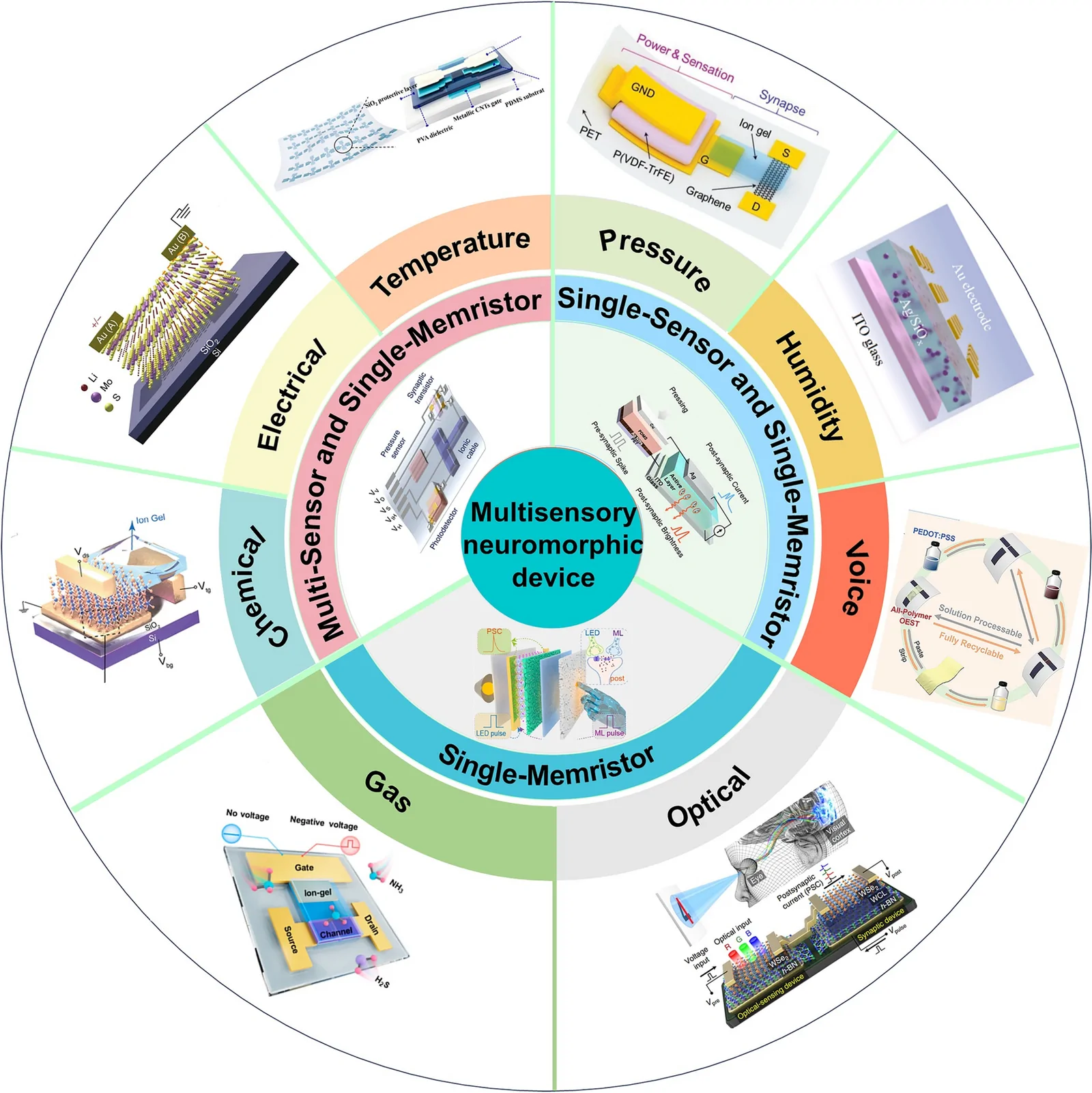
Schematic illustrating multisensory neuromorphic device with cross-modal stimuli integration and three multisensory fusion frameworks

In this study, multimodal specifically denotes the intrinsic fusion of heterogeneous physical stimuli into unified electrical representations at the device level, where a single device or array concurrently responds to multiple stimulus modalities (e.g., optical/electrical/thermal/pressure) [39]. This term describes hardware-centric capabilities, exemplified by phase-change materials simultaneously encoding diverse inputs. Multisensory describes bio-inspired system architectures that mimic neural integration of segregated sensory pathways [20]. This concept operates at the algorithmic/system level, utilizing neuromorphic computing principles (e.g., synaptic weight updates, spatiotemporal integration) to fuse signals into unified perceptual outputs, thereby emulating biological multisensory processing in the brain. Human multimodal perception integrates sensory information from various sources such as tactile, olfaction, hearing, and vision to make accurate judgments about object properties. Inspired by biological perception, neuromorphic systems based on multisensory memristors support efficient information integration and exhibit high fault tolerance. They are capable of perceiving multiple signals, including electrical, optical, pressure, voice, gas, humidity, temperature, and chemical signals (Fig. 1) [40–42].

The development of neuromorphic devices has the potential to overcome the limitations of the von Neumann architecture by mimicking the operation of biological brain function. The transmission, processing, and memorization of information in the human brain primarily depend on the intricate neuron network, comprising a vast number of neurons (approximately 10^11^) and their gapped junctions known as synapses (approximately 10^15^) [43]. Neurons serve as the fundamental units of brain function in humans, while synapses play a crucial role in enabling neurons to carry out signal transmission and information exchange [44, 45]. External information can be perceived and converted into chemical signals by neurons, and synapses facilitate the transmission of these signals from presynaptic neurons to postsynaptic neurons via neurotransmitters [46, 47]. Diverse external stimuli can influence the chemical fluxes within these synapses, thereby modulating the synaptic strength or weight.

In neuromorphic devices, synaptic plasticity induced by various input stimuli can have an impact on the construction of neuromorphic systems [48]. The simulation of biological synapses plasticity in neuromorphic devices is achieved by operating various resistance switching mechanisms [39, 49]. The switching mechanisms of neuromorphic include conductive filament, ion migration, charge trapping, electrochemical doping, phase transition, ferroelectricity, and other mechanisms [50–52]. The specific implementation methods of these resistance switching mechanisms depend on the materials and device structures used [53–55]. Understanding which synaptic mechanisms are more conducive to multisensory integration and what mechanisms and principles are involved in the fusion of multisensory signals is crucial for constructing efficient neuromorphic devices.

**Fig. 2:**
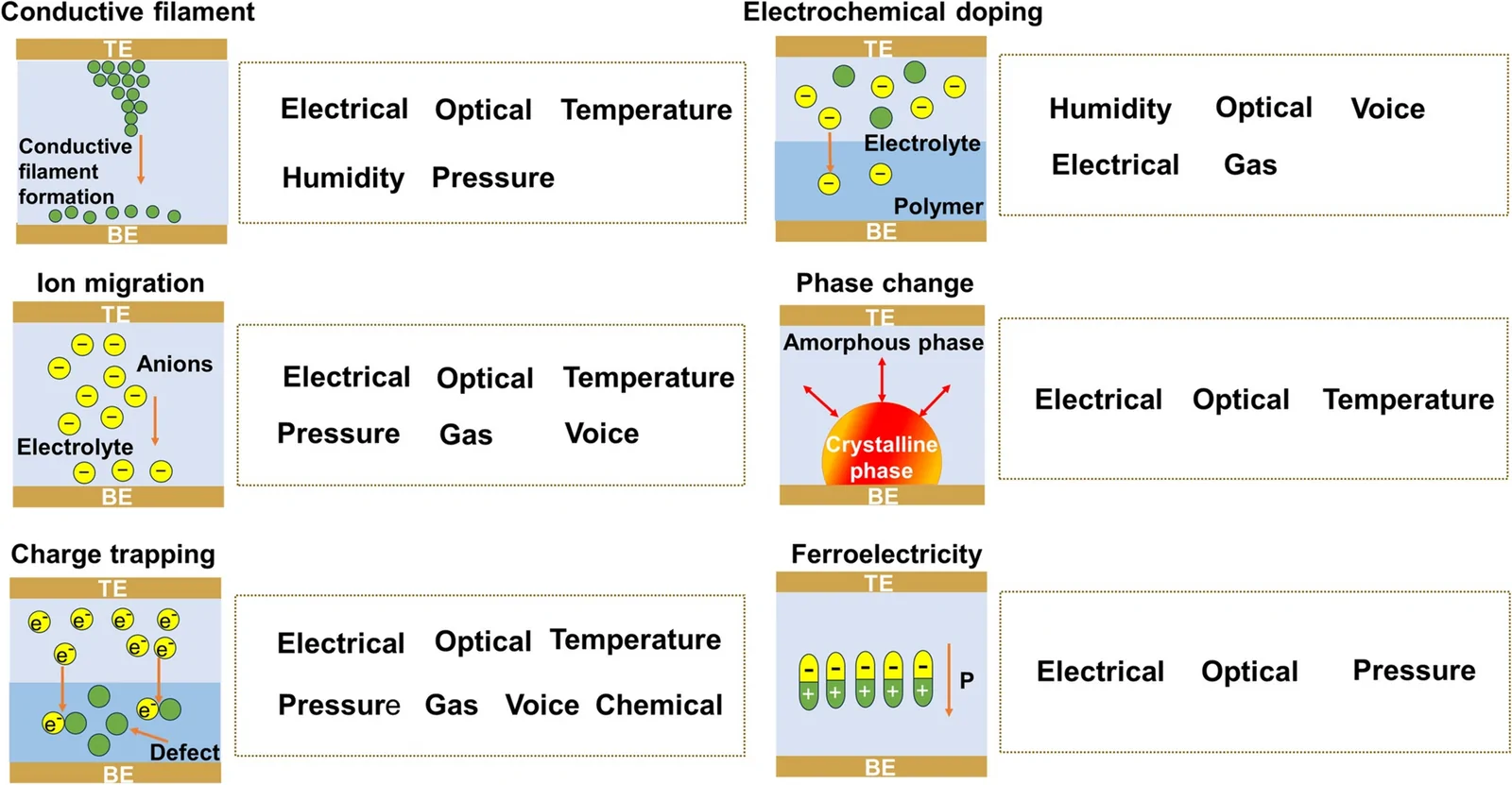
Physical mechanism mapping of neuromorphic devices to multimodal input stimuli

There are six different neuromorphic resistive switching physical mechanisms and their corresponding detectable input signals (Fig. 2). Among these six mechanisms, the conductive filament, ion migration, electrochemical doping, and charge trapping mechanisms can detect a wider variety of input signals. In contrast, the phase change and ferroelectric polarization mechanisms can detect fewer types of input signals on the right side of Fig. 2. Notably, the charge trapping mechanism can detect the largest variety of input signals, potentially making it more favorable for application in multimode neuromorphic devices. Charge trapping mechanism demonstrates the most extensive multimodal detection capability among the six mechanisms, primarily due to trap states' inherent sensitivity to diverse external stimuli [56]. Unlike mechanisms constrained by specific material phases, ion species, or lattice symmetries, this sensitivity universally arises at defective semiconductor/insulator interfaces or within bulk regions. The tunable nature of trap energy levels enables dynamic modulation by external inputs: light excitation generates electron–hole pairs that populate/deplete traps [57]; electric fields directly reconfigure trap occupancy [58]; chemical adsorption/reactions alter trap barriers via surface dipoles or charge transfer [59]; thermal energy governs shallow-trap carrier release for high-precision temperature response [60]; and mechanical strain expands trap capture cross-sections [61]. Crucially, all stimuli converge into a unified response paradigm that translates trap charge variations linearly or nonlinearly into measurable electrical signals through Schottky barrier height, capacitance, or resistance changes [62]. This intrinsic conversion of heterogeneous inputs into a single physical quantity allows charge trapping to function as a multimodal sensing front-end without requiring specialized crystal structures (e.g., phase-change materials) or ion migration pathways (e.g., electrochemical doping) [63]. Furthermore, traps originating from intrinsic defects, surface dangling bonds, interface states, or extrinsic dopants ensure compatibility with virtually any material system, including oxides, 2D materials, organic semiconductors, and perovskites [64]. This universality remains unattainable by other mechanisms constrained to specific material classes.

Phase change and ferroelectricity mechanisms detect the fewest signal types. Phase-change and ferroelectricity mechanisms exhibit the most limited signal perception capabilities due to unidimensional order parameter coupling and high activation thresholds [65, 66]. The order parameter of phase change is crystallinity, and phase change occurs only when the supplied energy exceeds the crystallization barrier [67]. It thus responds solely to heat accumulation and cannot directly couple to chemical or pressure stimuli. The order parameter of ferroelectricity is polarization, and polarization reversal demands overcoming the coercive field [68]. The ferroelectric mechanism exhibits exclusive sensitivity to electric field stimuli, while non-electric stimuli such as optical or pressure necessitate conversion into electric fields for effective perception [69, 70]. Conversely, charge trapping, ionic migration, and electrochemical doping mechanisms demonstrate direct, low-threshold responses to diverse stimuli (optical/chemical/electrical/pressure/temperature) through their order parameters (trapped charge, ion concentration, and redox states) [71–73]. Crucially, phase-change requires overcoming enthalpy of melting or lattice distortion energy, while ferroelectric switching demands high coercive fields. These energy barriers significantly exceed those for defect-level shifting, ion drift, or redox transitions. Consequently, stimuli beyond temperature and electricity rarely induce detectable changes within conventional energy ranges, inherently limiting perceptible signal diversity.

### Charge Trapping/De-trapping

**Fig. 3:**
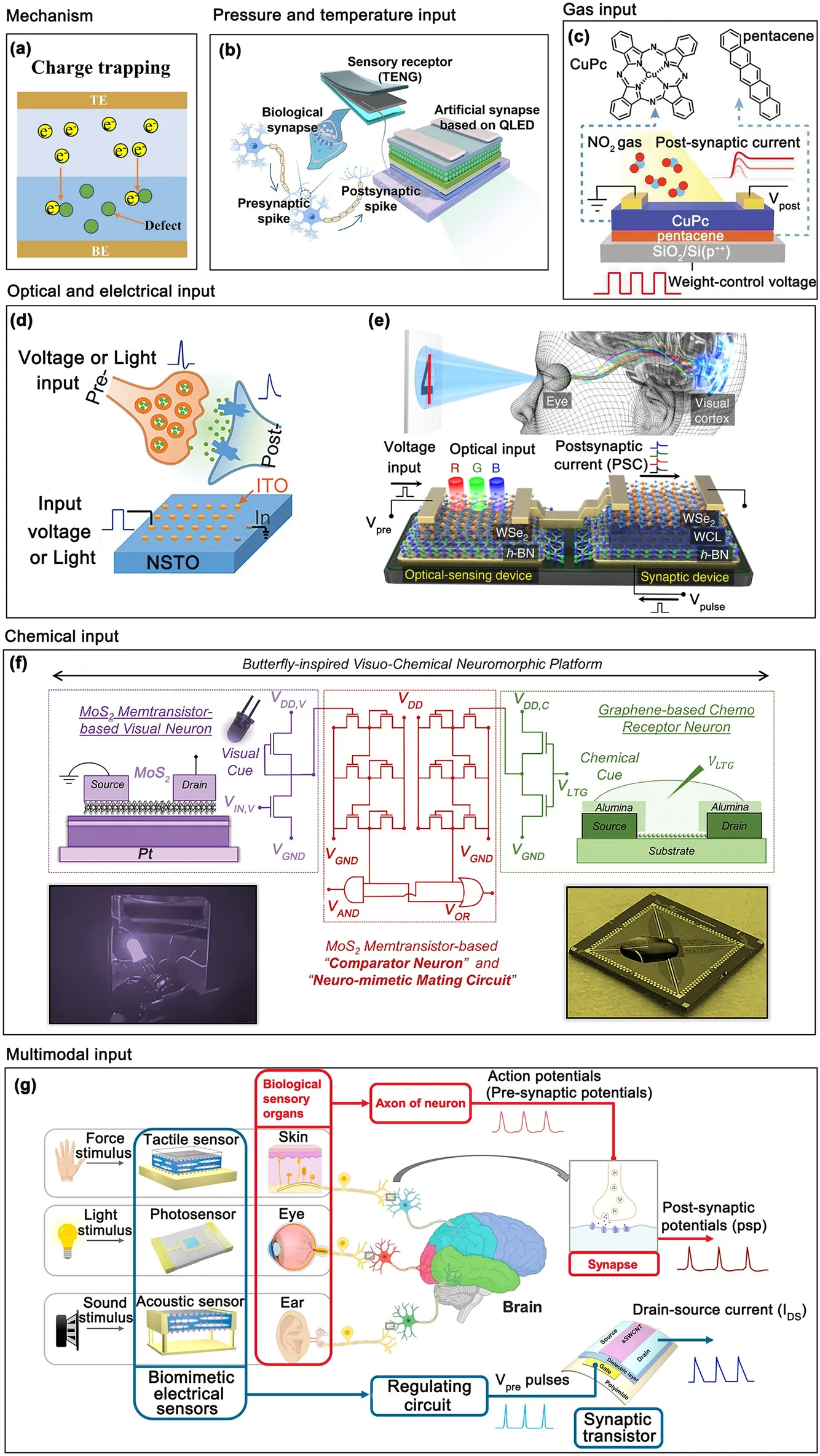
Charge trapping synaptic devices processing multimodal stimuli. **a** Resistive switching mechanism via defect trapping. **b** Artificial multimodal system schematic with pressure/temperature inputs. Reproduced with permission [92]. Copyright 2024, Chemical Engineering Journal. **c** Organic heterostructure sensory synapse for NO_2_ detection. Reproduced with permission [93]. Copyright 2022, Advanced Functional Materials. **d** Optoelectrical synapse experimental setup. Reproduced with permission [94]. Copyright 2024, Advanced Functional Materials. **e** Bio-inspired optoelectronic nerve system with h-BN/WSe_2_. Reproduced with permission [95]. Copyright 2018, Nature Communications. **f** Butterfly-inspired visuo-chemical neuromorphic platform using memtransistor circuits. Reproduced with permission [96]. Copyright 2024, Advanced Materials. **g** Biological sensory organs processing optical/pressure/voice stimuli and neural information transmission. Reproduced with permission [97]. Copyright 2021, ACS Nano

One prominent mechanism of resistive switching is charge trapping (Fig. 3a), which is mainly induced by four factors: defects caused by local structural distortion or dangling bonds [74, 75] defects at the interface between semiconductors and dielectrics [76–78] potential wells formed by a semiconductor bulk heterojunction [79, 80] and floating gates [81–83]. Charge trapping/de-trapping can generally adjusted by appropriate modulation of electrical or optical signals. Under an electric field, trapping and de-trapping can be controlled by applying and removing an electric field. Initially, due to the effect of an applied electric field, ion or vacancy defects are captured. Then, with a certain time delay, ion or vacancy defects are de-trapped when the electric field is removed or the direction of the applied electric field is reversed [84]. Under illumination, light energy is used to trigger the capture of photo-induced charges [85]. The light induced field generated by the captured charge promotes ion drift and diffusion, followed by applying a potential to achieve de-trapping [86]. These traps contribute to the slow decay of photocurrent in the device, and charge trapping and de-trapping can be used to provide controllable channel conductance modulation [87]. Due to the conductivity changes and stable and reversible physical operations that can occur during the charge trapping and de-trapping process, the charge trapping/de-trapping mechanism has been widely used to construct various memristors and neuromorphic devices [88, 89]. Charge trapping architectures implementations feature broad spectral response, exceptional endurance, and technological maturity [90]. Yet they suffer from slow write speeds and demand high-voltage operation [91].

Neuromorphic devices based on defect trapping mechanisms can perceive a variety of signals, such as optical, electrical, temperature, chemical, pressure, gas and voice signals. Chen et al. fabricated an artificial multimodal system that can sense pressure and thermal stimuli simultaneously and provide optical feedback (Fig. 3b) [92]. A triboelectric nanogenerator (TENG) is utilized as an artificial electronic skin to perceive pressure stimuli. Meanwhile, a quantum dot light-emitting diode (QLED) device serves as an artificial neuromorphic synapse, providing optical feedback and performing temperature sensing computation. The temperature sensing of the device is realized through the artificial light-emitting synapse, where the charge trapping layer captures charges affected by temperature, while the synaptic light-emitting is also influenced by temperature. As the temperature rises, the energy of the charges increases, making them less likely to be captured by traps, which in turn enhances the conductivity of the device. The pressure signal is generated as presynaptic spikes in an artificial light-emitting synaptic device by electrostatic induction and electrostatic equilibrium when the skin contacts and separates from polydimethylsiloxane (PDMS). Qian et al. developed an artificial sensory synapse for nitrogen dioxide (NO_2_) detection, which is composed of an organic heterostructure featuring a charge trapping layer and a hole-conducting layer (Fig. 3c) [93]. This artificial sensory synapse is capable of processing, assessing, and responding to different gaseous environments. NO_2_ permeates through the organic heterostructure and traps electrons in the charge trapping layer, leading to the accumulation of carriers in the hole-conducting layer and the retention behavior of the device. As learned above, neuromorphic devices can sense temperature, pressure, and gas signals through the defect trapping mechanism. For temperature signals, temperature changes affect the electrical and chemical properties of the material, altering defect trapping and release. For pressure signals, piezoelectric neuromorphic devices use piezoelectric potentials to modulate electrical transport, converting external mechanical motion into electrical signals and regulating synaptic weights. For gas signals, Gas signals alter surface charge distribution or chemical properties, influencing defect capture and release.

Instead of pressure, temperature and gas signals, some other external stimulation can also be sensed by charge trapping mechanism. Fang et al. fabricated an optoelectronic synaptic device of indium oxide (In_2_O_3_)·stannic anhydride (SnO_2_)/niobium-doped strontium titanate (Nb:SrTiO_3_) heterostructure, which vividly demonstrates the in-sensor computing capability and multimodal perception ability to sense both optical and electrical signals (Fig. 3d) [94]. The surface of Nb:SrTiO_3_ is abundant with interfacial defects dominated by oxygen vacancies, which facilitates the trapping/de-trapping of electrons. Under positive gate voltage or illumination, electrons trapped in the oxygen vacancies at the indium tin oxide (ITO)/Nb:SrTiO_3_ interface are released, leading to a decrease in the height and width of the Schottky barrier, and the device exhibits a low-resistance state (LRS). When the illumination is turned off or a negative gate voltage is applied, the electrons are recaptured by the oxygen vacancies, and the device exhibits a high-resistance state (HRS). Seo et al. reported a neuromorphic synaptic device with electrical and optical sensing functionalities, which fabricated on a hexagonal boron nitride (h-BN)/tungsten diselenide (WSe_2_) van der Waals (vdW) heterostructure (Fig. 3e) [95]. The working principle of the vdW synaptic device is based on the trapping/de-trapping of electrons within a weight control layer (WCL) on h-BN, which in turn modulates the conductivity of the WSe_2_ channel. Optical signals primarily regulate synaptic properties through wavelength modulation. Shorter optical wavelengths result in greater light absorption, which decreases the resistance of the synaptic device. Consequently, the density of carriers trapped within the WCL increases, thereby modulating synaptic properties. In contrast, electrical signals directly impact the carriers in the WCL, influencing their trapping/de-trapping processes. As learned above, photoelectric neuromorphic devices can sense optical and electrical signals through the defect trapping mechanism. For optical signals, the device converts optical signals to electrical signals. Photogenerated carriers are trapped or released at defect sites, altering the conductivity of device, thereby enabling the perception of optical signals. For electrical signals, neuromorphic devices use electrical stimuli to modulate defect trapping states, thereby adjusting the resistance or conductivity of device.

In addition to the aforementioned input signals, chemical and voice signals can also be sensed by the charge trapping mechanism. Zheng utilized the chemical sensing properties of graphene and the photo sensing capability of monolayer molybdenum disulfide (MoS_2_) to create a multimodal platform for visual–chemical integration (Fig. 3f) [96]. This device perceives chemical signals by deploying an artificial chemical receptor neuron consisting of two graphene chemical transistors connected in series. Aqueous solutions of chemicals are dripped onto the graphene channel area for chemical sensing. At the interface between the graphene channel and the chemical solution, an electrical double layer (EDL) is formed, which serves as an ultrafine dielectric layer. This EDL allows for the control of channel conductance when an electrical bias is applied to the solution, thereby controlling the carrier trapping/de-trapping process. Wan et al. proposed a multimodal artificial sensory memory system that possesses biomimetic sensory transduction, neurological capabilities, synaptic-like information processing, and memory functions (Fig. 3g) [97]. This system can perceive multiple signals, including optical, pressure, and voice signals. The multimodal perception of the system is achieved by utilizing polypropylene-based ferroelectret nanogenerator (FENG) as both tactile and acoustic sensors, along with phototransistors serving as optical sensors. Physical stimuli are converted into informational electric pulses, which are then transmitted through conditioning circuits to an artificial neural system for processing and storage. As learned above, neuromorphic devices can detect chemical and voice signals via defect trapping mechanisms. Chemical signals alter surface charge distribution or chemical properties, influencing defect trapping and release, and enabling the detection of chemical signals. Voice signals modulate the electrical polarization state of a material through the propagation of mechanical waves, thereby altering the electrical characteristics of the device.

Neuromorphic devices achieve multimodal perception through defect trapping mechanisms, where the diversity of defect types and their modality-specific interactions are critical [98, 99]. For instance, vacancy defects (e.g., sulfur vacancies in MoS_2_) dominate optical and electronical signals detection by modulating photogenerated carrier dynamics [100–102]. While surface defects enable chemical sensing via molecular adsorption at active sites [103, 104]. Grain boundary defects respond to mechanical stimuli such as pressure and voice through strain-induced polarization changes [105–107] and interface oxygen vacancies regulate temperature signals by altering phonon scattering pathways [108, 109]. Crucially, the spatial distribution and dynamic response characteristics of defects further enhance functionality [110]. Defects often form interconnected conductive networks (e.g., conductive filaments in memristors), where external signals differentially modulate localized pathway connectivity, enabling differentiated responses [42, 111]. The charge dynamics of defects depends on their energy levels [102, 112]. Shallow defects quickly trap and release charges (nanosecond timescales), making them suitable for detecting fast signals like sound or light pulses. Deep defects retain charges much longer (seconds to permanent states), enabling sustained responses to slow-changing signals such as temperature or steady pressure.

The transition from unimodal to multimodal perception stems from defect coupling and dynamic evolution. For example, sulfur vacancies in MoS_2_ can simultaneously respond to light and NO_2_ adsorption, enabling opto-chemical dual sensing [113, 114]. Moreover, defect distributions can dynamically evolve under external fields (e.g., voltage pulses inducing oxygen vacancy migration), allowing reconfigurable device functionality to adapt to multimodal switching [115]. This synergy of heterogeneous defect interactions and field-driven adaptability emulates biological sensory integration, offering a versatile platform for advanced neuromorphic systems.

### Ion Migration

**Fig. 4:**
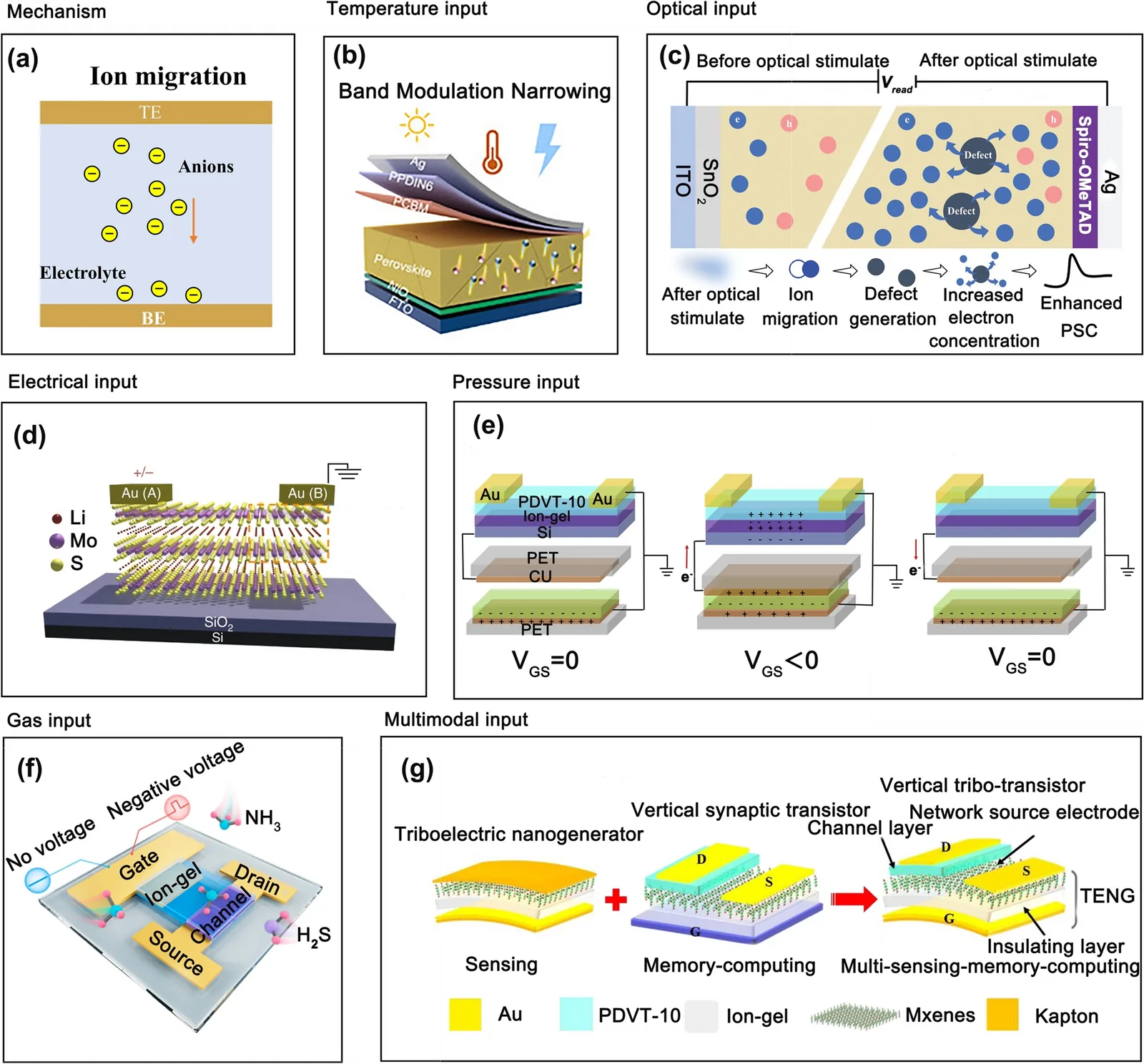
Ion migration synaptic devices processing multimodal stimuli. **a** Resistive switching mechanism via ion migration. **b** Perovskite photodetector schematic with optical/temperature/electrical stimuli. Reproduced with permission [134]. Copyright 2023, InfoMat.** c** Ion migration dynamics in perovskite devices under optical stimulation. Reproduced with permission [137]. Copyright 2024, Advanced Materials. **d** MoS_2_-based memristive device schematic with electrical stimuli. Reproduced with permission [138]. Copyright 2019, Nature Materials. **e** Self-powered synaptic transistor with pressure stimuli. Reproduced with permission [139]. Copyright 2019, Nano Energy. **f** Gas sensing OECT with embedded sensory function. Reproduced with permission [140]. Copyright 2024, ACS Sensors. **g** MXene tribo-transistor device integrating TENG and organic field-effect transistor for multimodal stimuli. Reproduced with permission [141]. Copyright 2022, Nature Communications

The ion migration mechanism in the resistive switching of neuromorphic devices is a crucial operational principle that emulates synaptic functionalities in biological neural networks, forming the foundation for neuromorphic computing. This mechanism refers to the directional migration of ions within the device under external stimuli, particularly electric fields, leading to changes in resistive states (Fig. 4a) [116, 117]. It typically involves ion diffusion and migration through solid materials, as well as interactions between ions and material defects, interfaces, and other structural features [118, 119]. This mechanism operates through ionic conduction without redox reactions, altering local charge distribution or forming conductive pathways solely via ion repositioning [120]. Architectures based on the ionic migration mechanism offer high-precision analog weighting with continuous conductance tuning at minimal power [121]. However, slow ion diffusion creates response latency, while environmental sensitivity compromises stability [122].

To implement the ion migration mechanism, materials with ionic conductivity must be selected. These materials commonly include ionic liquids, hydrogels, two-dimensional layered transition metal oxides (e.g., alpha-phase molybdenum trioxide (α-MoO_3_)), perovskites, and low-dimensional vdW crystals (e.g., niobium triselenide (NbSe_3_)) [123–125]. In these materials, ions migrate under applied electric fields [126]. Additionally, ion migration is closely linked to other resistive switching mechanisms, such as conductive filament formation, defect trapping, and electrochemical doping [127–129]. Neuromorphic devices often employ complex architectures to enable ion migration and resistive switching. These may incorporate multilayer structures, nanochannels, or discrete channels to provide pathways and spatial confinement for ion migration [130, 131]. By precisely regulating ion migration, fine-grained resistance control can be achieved, mimicking the complex synaptic weight modulation in biological neural networks. However, the ion migration mechanism may be influenced by factors such as material stability, ion diffusion rates, and device architecture [132, 133].

Neuromorphic devices based on ion migration mechanisms can perceive a variety of signals, such as optical, electrical, temperature, pressure, gas and voice signals. Li et al. fabricated a perovskite photodetector and proposed a novel strategy leveraging intrinsic ion migration in perovskites to construct narrow-band photodetection (Fig. 4b) [134]. By employing optical, temperature and electrical signals to manipulate ion migration, the band structure of the perovskite photodetector can be modulated in situ, thereby enabling precise regulation of its spectral response characteristics [135, 136]. The influence of temperature on ion migration mechanisms primarily manifests as enhanced conductivity and reduced band bending with increasing temperature. Elevated temperatures promote more uniform ion distribution, which diminishes interfacial ion accumulation and suppresses photogenerated carrier loss. Furthermore, at higher temperatures, thermally excited electrons transition from the valence band to the conduction band, thereby weakening the doping effects induced by ionic accumulation. Increased diffusion coefficients also facilitate rapid ion diffusion back into the perovskite bulk, effectively reducing trap states and improving carrier collection efficiency. Guo et al. proposed a two-terminal synaptic device based on lead halide perovskite, featuring in situ tunable optoelectronic properties (Fig. 4c) [137]. Optical signals can reduce the ion migration activation energy. Upon light stimulation, an appropriate amount of Br⁻ ions begin to migrate under the influence of voltage​, introducing corresponding donor levels. This migration increases the electron concentration and subsequently inducing stable enhancement of the postsynaptic current. Zhu et al. fabricated MoS_2_-based memristive devices and achieved reversible modulation of MoS_2_ films by controlling the migration of Li ions with sensed electrical signal, a process consistent with local 2H-1T' phase transitions (Fig. 4d) [138]. In this system, localized increases/decreases in Li ion concentration drive phase transformations between the 2H phase and 1T' phase. The engineered devices exhibit exceptional memristive behavior, enabling direct inter-device coupling via localized ionic exchange that inherently reproduces biological synaptic competition and cooperation effects. As learned above, neuromorphic devices can detect optical, electrical and temperature signals via ion migration mechanisms. Optical signals induce ion migration through photogenerated electric fields or photo-thermal effects, such as photo-induced oxygen vacancy migration. Electrical signals, on the other hand, modulate ion distribution via externally applied electric fields, as demonstrated by the formation and rupture of conductive filaments in memristors. On the other hand, thermal signals facilitate ion migration by providing the necessary energy through thermal activation processes.

In addition to the electrical and optical signals, pressure and gas signals can also be sensed by the ion migration mechanism. Liu et al. reported a novel self-powered synapse transistor by coupling an electric-double-layer organic field effect transistor and a TENG to sense pressure signal (Fig. 4e) [139]. Adjusting the distance between two electrodes of TENG generates varying voltages, which serve as presynaptic spikes. Before combining with a memristor synapse, TENG induces net positive charges on the bottom Cu film and net negative charges on the PDMS film. External touch on TENG brings the top Cu film into contact with the bottom PDMS film. At this point, electrons flow from the top Cu film to the silicon (Si) gate via electrostatic induction, leaving the Cu film with net positive charges and the Si gate with net negative charges, thereby creating a negative gate voltage in the transistor device. Simultaneously, under EDL effects, cations and anions accumulate at the Si/Ion-gel and Ion-gel/poly[2,5-bis(alkyl)pyrrolo[3,4-c]pyrrolo-1,4(2H, 5H)-dione-alt-5,5-di(thiophene-2-yl)-2,2-(E)-2-(2-(thiophen-2-yl)vinyl)thiophe4ane] (PDVT-10) interfaces, respectively. This induces positive charges on PDVT-10 at the PDVT-10/Ion-gel interface, enhancing channel carrier density and channel current, ultimately leading to increased excitatory postsynaptic current (EPSC). Yin et al. presented a gas sensing organic electrochemical transistor (OECT) embedded with sensory functionality, demonstrating integrated capabilities including chemical information decoding, tunable memory states, and gas sensing selectivity (Fig. 4f) [140]. The ion-gel electrolyte endows the device with tunable memory characteristics and low operational voltage, while enabling the realization of essential synaptic behaviors such as short-term plasticity and paired-pulse facilitation (PPF). The ion-gel electrolyte mitigates gas molecule adsorption/desorption in the semiconductor layer, thus enhancing the retention of gas sensing information. Typically, free ions in the ion-gel bind with ammonia (NH_3_) molecules. In the absence of a gate voltage, NH_3_ remains bound to the semiconductor surface. When a negative gate voltage is applied, both the ions and their bound NH_3_ migrate into the bulk semiconductor, enabling dynamic erasure of stored gas signals through voltage-driven ionic redistribution. As discussed above, neuromorphic devices can detect pressure and gas signals through ion migration mechanisms. For gas signals, molecular adsorption modulates the ion migration barrier by altering surface charge states or chemical potentials. For pressure signals, mechanical stress generates localized electric fields via the piezoelectric effect, converting mechanical vibrations into electrical signals that drive directional ion migration.

Simultaneously, multimodal recognition can be achieved through ion migration mechanisms. Liu et al. developed a Ti_3_C_2_T_x_ MXene-based vertical tribo-transistor device integrating a TENG and a vertical organic field-effect transistor, capable of multimodal memory-computing functions and multimodal affective recognition for optical, pressure, and voice signals (Fig. 4g) [141]. The sensing capabilities of vertical tribo-transistor are emulated through the actuation of the gate electrode and device vibrations, enabling multimodal perception of pressure and voice signals, while optical signal detection is achieved via a photosensitive MXene electrode. Charges accumulate in the TENG through electrostatic induction and triboelectric charging. The resulting triboelectric potential dynamically modulates ion migration within the dielectric layer and adjusts the Schottky barrier height at the MXene/semiconductor interface, thereby regulating the conductive channel between the MXene and drain electrode. The device extracts discriminative features from optical and voice signal modalities and relays this information to the input layer for advanced processing, employing data-level fusion to integrate feature sets derived from multiple sensory channels. This synergistic combination of cross-modal features enhances both the accuracy and robustness of perceptual recognition, emulating biological multisensory integration mechanisms observed in neural systems.

The core mechanism enabling neuromorphic devices to achieve multimodal perception through ion migration lies in the high sensitivity of ion migration to multiphysical field stimuli and its dynamic re-configurability [36, 39, 127]. This capability lies in a unified physical mechanism of ion motion, which transduces input signals from various energy modalities (optical, electrical, temperature, chemical, pressure, etc.) into nonlinear conductance or resistance variations, thereby mimicking the plasticity of biological synapses [142, 143]. Crucially, ion migration exhibits intrinsic multiphysical coupling: ionic motion responds sensitively to multiple energy modalities, functioning as a natural multimodal signal transducer [144]. All external signals ultimately modulate material conductivity by altering ion migration rates or pathways, enabling cross-modal signal conversion [145, 146]. The dynamic re-configurability of ion migration further enhances functionality, as external fields (e.g., electric and optical) can realign ion migration paths and spatial distributions in real time, endowing devices with adaptive perception and learning capabilities [147, 148]. For instance, the synergistic control of light intensity and electric fields can regulate oxygen vacancy migration in oxide materials, enabling on-demand switching between optical and electrical sensing modes [149, 150]. This re-configurability mirrors ability of biological systems to prioritize sensory inputs based on environmental context, laying the foundation for context-aware neuromorphic computing.

### Conductive Filament

**Fig. 5:**
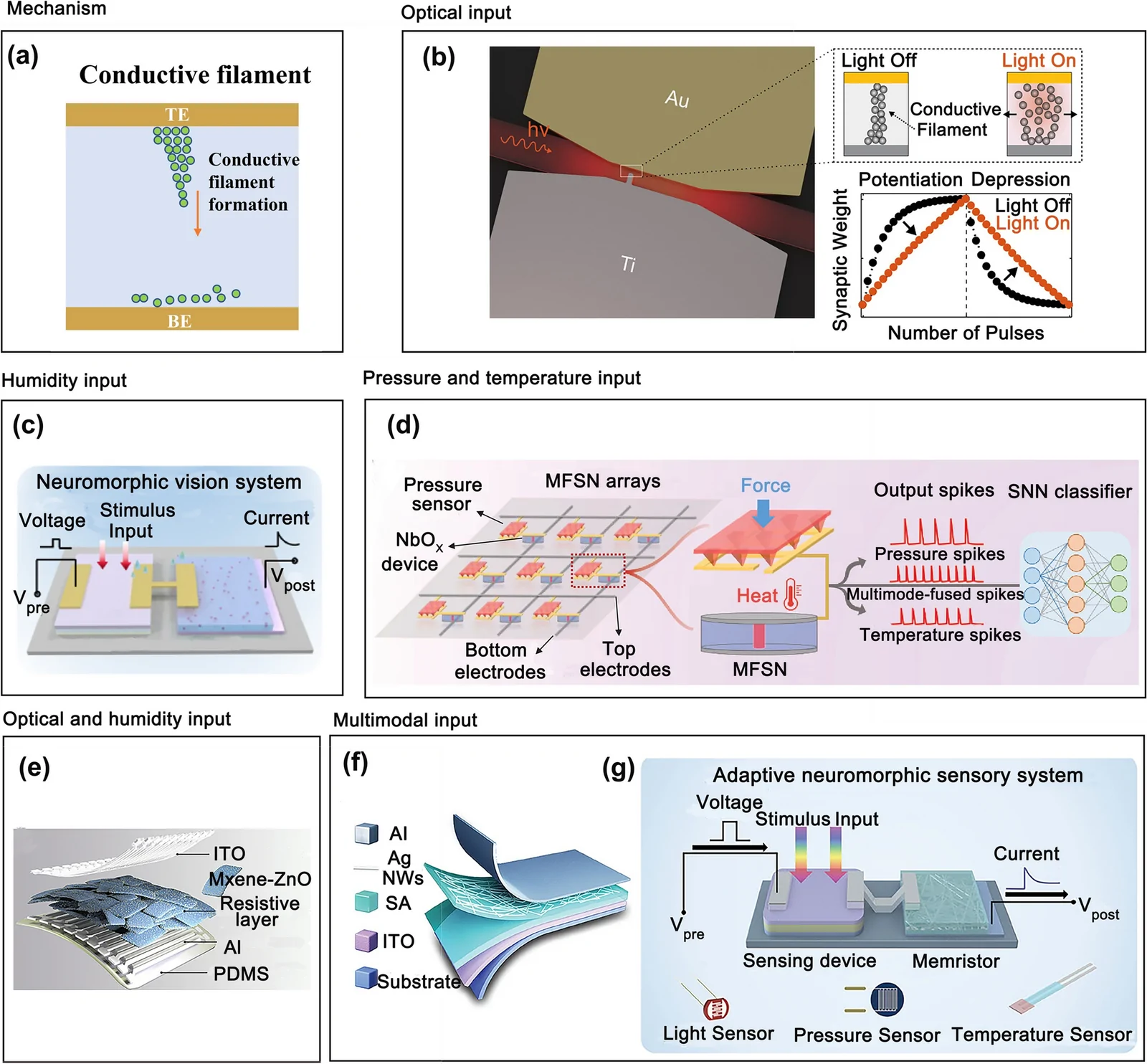
Conductive filament synaptic devices processing multimodal stimuli. **a** Resistive switching mechanism via conductive filament formation. **b** Plasmonic Au-HfO_2_-Ti waveguide schematic with optical input. Reproduced with permission [161]. Copyright 2021, ACS Nano. **c** Neuromorphic vision units for humidity perception. Reproduced with permission [162]. Copyright 2024, Materials Today Nano. **d** Artificial somatosensory system with MFSN array processing pressure/temperature stimuli. Reproduced with permission [163]. Copyright 2022, Advanced Materials. **e** Flexible MXene-ZnO memristor with optical/humidity stimuli. Reproduced with permission [164]. Copyright 2021, Advanced Functional Materials. **f** Ag NW-embedded SA memristive device structure. Reproduced with permission [41]. Copyright 2024, Advanced Materials. **g** Adaptive neuromorphic sensory units for multimodal perception

The conductive filament mechanism is one of the core physical mechanisms for achieving resistance regulation in neuromorphic devices (Fig. 5a). It simulates synaptic weight changes in biological systems through the dynamic formation and rupture of microscopic conductive paths, providing hardware foundations for brain-inspired computing and memory-computing integrated systems [151, 152]. The formation and rupture of conductive filaments are considered to be caused by electric field-driven ion migration and redox reactions [50]. Depending on the types of mobile ions, the mechanisms can be classified into two categories: electrochemical metallization (ECM) and valence change mechanism (VCM) [33].

In ECM memristors, the formation and rupture of conductive filaments primarily rely on redox reactions of active metals [36]. These memristors typically consist of active metal electrodes (e.g., Ag, Cu), inert metal electrodes (e.g., Pt, Au), and dielectric layers (e.g., silicon dioxide (SiO_2_), tantalum oxide (TaO_x_)) [153]. When voltage is applied across the electrodes, redox reactions occur in the active metal, generating metal ions that migrate through the insulating layer under the electric field, ultimately forming conductive filaments and resulting in a low-resistance state [154]. Upon applying reverse voltage, metal atoms in the filaments are oxidized into ions that return to the original electrode, or the filaments are melted through Joule heating effects, causing path interruption and restoring the device to a high-resistance state [155]. In VCM memristors, the formation and rupture of conductive filaments are mainly influenced by electric fields and anion migration [156]. When voltage is applied, the electric field drives anion migration (e.g., oxygen ions) within the material. This migration induces valence changes in cations, thereby forming or breaking conductive filaments. The conductive filament mechanism enables multi-level resistance state switching and achieves lower power consumption in devices [157]. Architectures based on the conductive filament mechanism achieve ultralow-voltage operation through simple two-terminal structures, with dynamically adjustable filaments naturally suited for neuromorphic computing [158]. But stochastic filament growth causes dispersed conductance distributions and poor endurance [159].

The filament formation and rupture processes are affected by multiple factors including material properties and various external stimulus such as electrical, optical, pressure, temperature and humidity signals [128, 160]. Portner et al. integrated dual-terminal valence change memory devices into photonic/plasmonic circuitry and demonstrated that switching characteristics of the memristor can be optically modulated (Fig. 5b) [161]. The added fiber-optic input serves as a third independent modulation channel for the device. The operational mechanism of device relies on localized photo-induced heating within the VCM mechanism. This localized thermal excitation enhances oxygen vacancy generation in the active region. Heating governs the lateral expansion of conductive filaments composed of oxygen vacancies by modulating the generation/recombination rate and diffusion dynamics of conductive filaments. This optothermal synergy thereby achieves more linear and symmetric switching characteristics under optical illumination. Han et al. demonstrated a multimodal neuromorphic sensory system based on Ag loaded porous silicon oxide (SiO_x_) based memristor, which exhibits highly controllable potentiation/depression characteristics modulated by relative humidity conditions (Fig. 5c) [162]. Physical mechanism analysis reveals that high relative humidity environments induce accelerated ion diffusion, thereby promoting conductive filament formation. The engineered synaptic memristor successfully emulates biological behaviors such as EPSC and PPF. As discussed above, neuromorphic devices can detect electrical, optical and humidity signals through conductive filament mechanisms. Electrical signals directly induce ion migration or metallic electrodeposition through applied electric fields to form conductive filaments. Optical signals accelerate filament growth by generating photogenerated electron–hole pairs or via photo-thermal effects that reduce ion migration energy barriers. Meanwhile, humidity signals modulate ion mobility through water molecule permeation, thereby altering the formation threshold of conductive filaments.

In addition to the electrical, optical and humidity signals, pressure and temperature signals can also be sensed by the conductive filament mechanism. Zhu et al. presented an artificial multimodal sensory system comprising a multimodal fusion spiking neuron (MFSN) array operating in the spiking domain and a spiking neural network (SNN) classifier (Fig. 5d) [163]. This system processes temperature and pressure multimodal inputs while preserving unimodal information fidelity. Each MFSN unit integrates a piezoresistive pressure sensor and a niobium oxide (NbO_x_)-based threshold switching memristor exhibiting temperature-dependent switching characteristics. When subjected to varying pressure intensities, the MFSN unit transduces mechanical stimuli into spikes with frequency-encoded pressure information. Concurrently, temperature fluctuations modulate threshold voltage of the memristor, inducing amplitude- and frequency-variant spike outputs. This enables decoupled extraction of pressure/temperature information through distinct spike frequency and amplitude signatures for multimodal tactile perception. Furthermore, under concurrent pressure–temperature stimuli, the MFSN unit encodes both modalities into unified spike trains, demonstrating efficient data compression capabilities through temporal multiplexing. Wang et al. designed and engineered a multimodal MXene-zinc oxide (ZnO) memristor that synergistically merges optical signal sensing, relative humidity signal sensing, and in-sensor preprocessing functionalities to emulate the environment adaptive behaviors unique to the human eye (Fig. 5e) [164]. The multifield-controlled resistive switching in this MXene-ZnO memristor originates from the photon/proton-regulated formation of oxygen vacancy filaments. Under high-humidity conditions, water molecules adsorb onto the MXene-ZnO heterojunction through dual hydrogen bonding. Hydrolysis of surface functional groups elevates ionic conductivity, thereby enhancing the humidity sensitivity of the MXene-ZnO system. When a negative voltage is applied to the device, oxygen vacancies form conductive filaments by migration. The impact of humidity signals on the device mainly lies in the suppression of resistive switching in memristors under high humidity conditions. The absorption of UV photons with energies exceeding the ZnO bandgap generates excitons at the MXene-ZnO heterostructure interface, followed by their dissociation. The liberated photoelectrons are captured by MXene, establishing an internal electric field. The influence of optical signal on the device primarily arises from the photogating effect, which induces the formation of oxygen vacancy filaments and subsequently governs light-mediated resistive switching. As discussed above, neuromorphic devices can detect temperature and pressure signals through conductive filament mechanisms. Temperature signals modulate the activation energy required for ion migration through thermal excitation, thereby altering the formation threshold of conductive filaments. Pressure signals regulate filament connectivity via piezoelectric effects or geometric deformation-induced lattice strain.

Simultaneously, multimodal recognition can be achieved through conductive filament mechanisms. Shi et al. emulated sensory adaptation functionalities through complementary switching in sodium alginate-based memristors embedded with silver nanowires, enabling multimodal perception capabilities that process optical, temperature, and pressure signals (Fig. 5f) [41]. Three types of adaptive neuromorphic sensory systems are constructed to achieve diverse perceptual modalities by integrating sensors with complementary memristors (Fig. 5g). Once the sensor detects environmental stimuli, the resistance of the sensor decreases, causing the voltage drop across the sensor to decrease, thereby increasing the voltage applied to the memristor since the sensor and memristor are connected in series. Then, the high-voltage excitation can switch the memristor. Functioning as an adaptive signal processor, the memristor dynamically modulates the electrical signals transduced by the sensor from environmental stimuli, emulating biological synaptic plasticity through its tunable conductance states.

There exist fundamental differences between ECM and VCM conductive filament memristors in their sensing mechanisms and multimodal signal processing capabilities. These distinctions originate from their disparate physical mechanisms and material properties. ECM relies on the electrochemical deposition of metal ions, while VCM is based on valence transitions of oxygen vacancies. For pressure response, mechanical stress directly distorts the metallic conductive filament path in ECM, causing abrupt resistance changes, whereas oxygen vacancy channels in VCM mechanisms are minimally affected by stress [165]. ECM's perception of optical signals is indirect. For optical signal detection, the ECM mechanism requires an additional photosensitive layer to generate photogenerated carriers that alter the interfacial electric field, thereby driving metal ion migration and inducing resistance state changes [157]. The VCM mechanism responds directly to optical signals. Light signals excite oxygen vacancy ionization, increasing vacancy concentration to directly modulate filament resistance [166]. For electrical signal perception, the ECM mechanism exhibits higher sensitivity to electrical signals, as filament formation/rupture is directly voltage-controlled [167]. Regarding temperature sensing, temperature influences filament rupture in ECM and migration speed of oxygen vacancies in VCM [168, 169]. ECM demonstrates greater temperature sensitivity with significant changes near room temperature, while VCM requires elevated temperature ranges for observable effects.

The sensing variable of ECM conductive filaments is the alteration of metal ion migration barriers, requiring external algorithms to distinguish different stimuli [170]. Device implementation of multimodal signal perception generally requires auxiliary external circuits, such as photodetectors and temperature sensors. In contrast, the sensing variable of VCM conductive filaments is the change in oxygen vacancy concentration/diffusion coefficient, leveraging sensitivity differences to various stimuli and multi-level weights to directly decouple signals at the device level [171]. The device is natively compatible with electrical, optical, and thermal stimuli, enabling multimodal fusion within a single device.

The core principle enabling neuromorphic devices to achieve multimodal perception (optical, electrical, temperature, humidity, pressure, etc.) through conductive filament mechanisms lies in their unified physical process (the dynamic formation/rupture of conductive filaments). This process provides highly sensitive responses to diverse physical signals while integrating nonlinearity, dynamic re-configurability, and brain-like characteristics [39, 172]. Conductive filament formation and rupture can be regulated by distinct physical fields, allowing all signals to be transduced into resistance changes via the same dynamic filamentary process [160, 164]. This unification simplifies hardware design for multimodal signal fusion and enables perception-computation integration. Furthermore, filament formation requires overcoming critical energy thresholds (e.g., voltage, light intensity, or temperature), exhibiting nonlinear switching behavior that closely mimics the action potential triggering in biological neurons [173]. The morphology of conductive filaments (length, branching, and density) can be dynamically reprogrammed in real time via external fields (e.g., electric pulses and light patterns), enabling functional switching and adaptive perception [174]. Through material design and external field modulation, selective signal response and synergistic enhancement can be achieved [175]. This unique combination of universal signal transduction, bio-inspired nonlinearity, and field-programmable adaptability positions conductive filament mechanisms as an ideal carrier for neuromorphic systems that emulate biological multisensory integration.

### Ferroelectricity

**Fig. 6:**
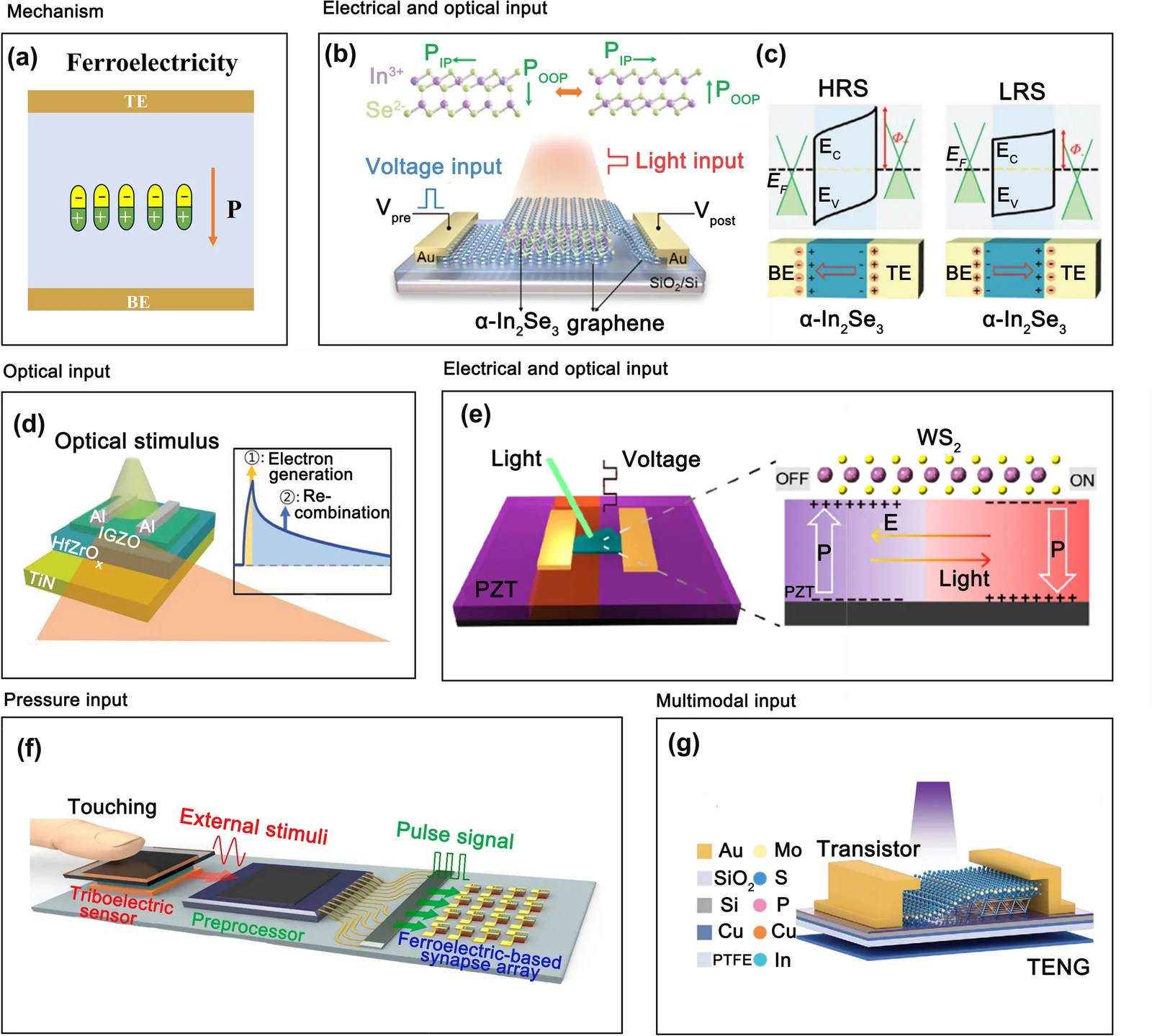
Ferroelectric synaptic devices processing multimodal stimuli. **a** Resistive switching mechanism via ferroelectric polarization. **b** In_2_Se_3_ ferroelectric synaptic device with electrical/optical inputs. Reproduced with permission [191]. Copyright 2024, Advanced Functional Materials. **c** Polarization-dependent band diagram of device in **b**. **d** Photonic synapse structure with IGZO transistors. Reproduced with permission [192]. Copyright 2020, Advanced Materials.** e** Optically/electrically tunable channel conductance mechanism. Reproduced with permission [202]. Copyright 2020, ACS Nano. **f** Neuromorphic pressure perception system. Reproduced with permission [197]. Copyright 2023, ACS Nano. **g** 3D asymmetric MoS_2_/CuInP_2_S_6_ synaptic heterostructure with multimodal stimuli. Reproduced with permission [198]. Copyright 2024, Advanced Functional Materials

The ferroelectricity mechanism is a resistive switching mechanism based on the reversible control of spontaneous polarization orientation in ferroelectric materials (Fig. 6a) [176, 177]. Electrically regulating the polarization states of ferroelectric domains mimics the synaptic weight modulation in biological synapses. This approach offers several advantages such as non-volatility, low power consumption, and high endurance, making it one of the core solutions for constructing high-performance neuromorphic devices [178, 179]. Ferroelectric materials (e.g., hafnium dioxide (HfO_2_), lead zirconate titanate (PZT), bismuth ferrite (BiFeO_3_)) exhibit spontaneous polarization whose direction can be reversed by external electric fields [180, 181]. Changes in polarization states directly influence the internal band structure, carrier distribution, and interface barriers of the material, thereby modulating device resistance [182, 183]. Ferroelectric architectures enable wear-free polarization switching for nanosecond operations and ultralow power consumption [184]. Yet they suffer from retention decay due to fatigue and interfacial defects, alongside unstable ferroelectricity at nanoscale [185].

When polarization aligns with the electric field, the induced interface barrier lowers with enhanced carrier injection, reducing resistance [186]. Conversely, when the electric field direction reverses, polarization inversion elevates interface barriers, impedes carrier transport, and increases resistance [187]. Ferroelectric memristors can also achieve continuously tunable intermediate resistance states through partial polarization reversal (metastable domain configurations), emulating the gradual modulation of synaptic weights [188, 189]. However, depolarization phenomena remain a common issue in ferroelectric memory and may cause drift and instability in resistance states within neuromorphic devices [32, 190].

The ferroelectric polarization of neuromorphic devices is influenced by various factors, including material properties and external stimuli such as electrical, optical, and pressure signals. Zeng et al. proposed a multimodal artificial synapse featuring a crossbar structure composed of graphene/alpha-indium selenide (*α*-In_2_Se_3_)/graphene layers, which can sense optical and electrical signals (Fig. 6b) [191]. The device integrates sensing, memory, and computing while mimicking various synaptic characteristics. Ferroelectric polarization modulates the Fermi level of graphene, thereby manipulating the asymmetric energy band alignment and inducing asymmetric contact barrier modulation (Fig. 6c). When the polarization is oriented downward, charge accumulation at the interface causes the Fermi level of the top graphene layer to shift downward, slightly increasing the contact barrier height and establishing a HRS. Conversely, upward polarization shifts Fermi level of the graphene upward, reducing the contact barrier height and resulting in a LRS. Kim et al. fabricated a photonic synaptic device with optically tunable synaptic plasticity by integrating oxide semiconductors and ferroelectric materials (Fig. 6d) [192]. The photoresponse characteristics of indium gallium zinc oxide (IGZO) were investigated under optical stimulation. Optical excitation enhances the conductivity of IGZO, while the channel conductance gradually decays over time upon stimulus removal. Under downward polarization, spatial separation between photogenerated electrons and ionized oxygen vacancies suppresses recombination processes, thereby prolonging the relaxation time. Conversely, upward polarization induces electron accumulation at the IGZO/ferroelectric layer interface, accelerating conductance decay. This demonstrates that ferroelectric polarization in the interfacial layer can be strategically employed to tailor the relaxation dynamics of oxide semiconductor-based photonic synapses. Luo et al. report a ferroelectric field-effect memtransistor for optoelectronic synaptic devices, fabricated using a two-dimensional tungsten disulfide (WS_2_) semiconductor on a ferroelectric PZT thin film (Fig. 6e) [153]. The WS_2_ channel exhibits electrically and optically controlled memristive switching, governed by the optically and electrically tunable ferroelectric domain configurations in the underlying PZT layer. When the PZT is in an upward polarization state, photoexcitation in WS_2_ generates intralayer excitons that decay into interlayer excitons, leading to positive charge accumulation at the WS_2_/PZT interface. These photo-induced charges screen the upward polarization and trigger polarization reversal.

As learned above, photoelectric neuromorphic devices can sense optical and electrical signals through the ferroelectricity mechanism. The principle of electrical signal detection in ferroelectric devices primarily relies on the non-volatile electric field control of ferroelectric polarization, wherein domain switching (reversal of polarization vector P) occurs when an external electric field exceeds the coercive field strength [193]. The principle of optical signal detection in ferroelectric devices is primarily the pyroelectric effect and photo-induced depolarization. The pyroelectric effect refers to light irradiation causing temperature changes in the material, leading to alterations in spontaneous polarization strength [194]. Photo-induced depolarization constitutes the main principle for optical signal detection in ferroelectric devices. Photo-induced depolarization in ferroelectric materials denotes the physical process where spontaneous polarization strength decreases or even vanishes under illumination, with its essence being that photon energy disrupts the ordering of spontaneous polarization in ferroelectrics [195]. Light excitation promotes valence band electrons to the conduction band, generating electron–hole pairs. These free carriers migrate under electric fields, screening ferroelectric polarization charges (e.g., compensating surface-bound charges), thereby weakening macroscopic polarization intensity [196]. Photon energy may also induce local lattice expansion, triggering a transition from ferroelectric to paraelectric phase, causing polarization direction rotation or disappearance [69]. Simultaneously, at the ferroelectric/electrode interface, redistribution of photogenerated charges at Schottky barriers alters the interfacial electric field, thus further weakening or reversing macroscopic polarization [195].

Simultaneously, pressure signal sensing and multimodal recognition can be achieved through ferroelectricity mechanism. Kim et al. proposed a tactile neuromorphic system for sensing pressure signals, which utilizes a triboelectric sensor based on PDMS and an ferroelectric synapse based on a MoS_2_/poly(vinylidene fluoride-trifluoroethylene) (P(VDF-TrFE)) heterostructure (Fig. 6f) [197]. The triboelectric sensor simulates the human tactile organs by converting pressure signal into electrical signals in real-time. Gong et al. presented a multimodal mechano-photonic synaptic memory device based on an asymmetric ferroelectric heterostructure, capable of cooperative modulation through external optical signals and pressure stimuli (Fig. 6g) [198]. The artificial synaptic architecture integrates an asymmetric MoS_2_/copper indium thiophosphate (CuInP_2_S_6_) ferroelectric hetero-field-effect transistor with a TENG unit that supplies triboelectric potentials for gating, programming, and plasticity control. Under triboelectric potential modulation, the device demonstrates exceptional mechanical displacement-derived electrical properties. Simultaneously, optical inputs trigger postsynaptic currents and update synaptic weights, successfully achieving cooperative modulation of triboelectric potentials and mechanical plasticity. This synergy enables the implementation of multimodal spatiotemporally correlated dynamic logic operations. The principle of pressure signal detection in ferroelectric devices is mainly the piezoelectric effect. The piezoelectric effect of ferroelectric materials serves as the physical foundation for pressure sensing: when mechanical stress acts on the device, lattice deformation causes rearrangement of electric dipoles, inducing changes in surface-bound charges (positive piezoelectric effect) [199].

The primary reason why neuromorphic devices achieve multimodal perception (optical, electrical, pressure, etc.) through ferroelectricity mechanisms lies in the multiphysical field coupling capability of ferroelectric materials and the homogeneous modulation characteristics of polarization dynamics [39, 127]. The spontaneous polarization orientation in ferroelectric materials can be directly or indirectly regulated by multiple physical fields (e.g., light, electric fields, and pressure), forming a unified signal transduction mechanism [200]. All external stimuli are converted into electrical responses (resistance and capacitance) through dynamic adjustments of polarization orientation or intensity, eliminating the need for discrete sensors and enabling hardware-level signal fusion [177, 201]. Furthermore, the ferroelectric polarization mechanism exhibits brain-like characteristics, such as non-volatile memory and nonlinear threshold responses, along with high energy efficiency and environmental robustness [183]. This intrinsic synergy between multiphysical adaptability and bio-inspired functionality positions ferroelectric materials as a transformative platform for neuromorphic systems requiring multimodal sensing-computing integration.

### Phase Change and Phase Transition Mechanism

**Fig. 7:**
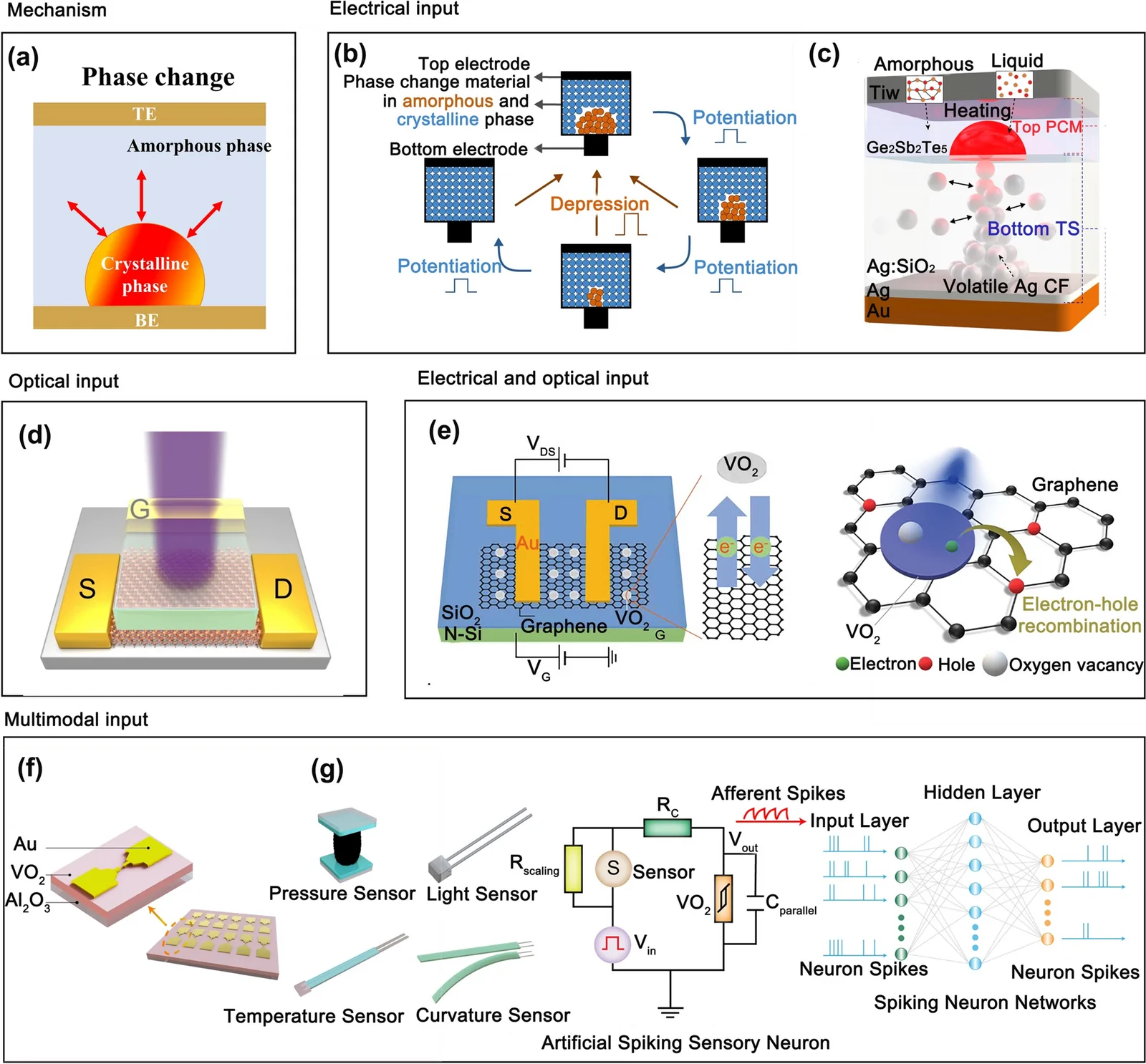
Phase-change synaptic devices processing multimodal stimuli. **a** Resistive switching mechanism via phase change. **b** PCM device structure with phase-change material between electrodes. Reproduced with permission [218]. Copyright 2018, Nature Communications. **c** PCM with volatile threshold switching and non-volatile PCM layer. Reproduced with permission [221]. Copyright 2022, Nature Communications. **d** Neuromorphic transistor under 375 nm UV stimulation. Reproduced with permission [224]. Copyright 2022, Nature Communications. **e** Au/VO_2_-graphene/Au photoelectric synapse with electrical/optical inputs. Reproduced with permission [237]. Copyright 2024, Advanced Functional Materials. **f** VO_2_ memristive device structure. Reproduced with permission [238]. Copyright 2022, Nature Communications. **g** Spike-based neuromorphic perception system for multimodal stimuli

Phase-change mechanism (PCM) is the reversible change of a system from amorphous state to crystalline state by Joule heating (Fig. 7a) [203–205]. When a sufficiently large electrical pulse is applied to generate the local heat exceeds the crystallization temperature, a crystallization can occur at the amorphous region. This is called as the “set” operation [206, 207]. In contrast, when the temperature exceeds the melting point of the substance, the crystalline region melts into an amorphous state. This is called as the “reset” operation [206, 207]. Due to the different bonding modes, the crystalline and amorphous phases exhibit distinct structures in terms of structural long-range ordering and periodicity, which results in unusual electrical and optical properties [208]. The amorphous state is a HRS, and the crystalline state is a LRS. The conventional phase change materials are the higher chalcogenides, such as tellurides and selenides [209, 210]. Tellurides and selenides are prone to transition between the amorphous and crystalline phases because of their low melting and crystallization temperature [211, 212]. The most widely studied of the tellurides and selenides are germanium-antimony-tellurium alloys, such as germanium-antimony-tellurium (Ge_2_Sb_2_Te_5_, commonly abbreviated as GST) [210, 213]. Phase-change architectures provide non-volatile memory with high cycling endurance and large resistive windows [214]. However, they require high operational energy and face speed limitations from thermal diffusion, while repeated phase changes accumulate grain boundary defects [215].

The phase-change mechanism primarily involves applying a sufficiently large electrical pulse to generate localized heat exceeding the crystallization temperature, inducing crystallization in the amorphous region, hence commonly used for sensing electrical signals (Fig. 7b, c). Using the difference in the resistance between amorphous and crystalline state, a series of synaptic functions can be mimicked with PCM. The Joule heating that induces the phase transition generally comes from two different sources. One is that heating is performed directly inside the phase change material [216–220]. Boybat et al. realized a synaptic device based on GST phase-change material, which consists of a layer of phase change material and two metal electrodes (Fig. 7b) [218]. When a current pulse of sufficient intensity is applied to phase change material, the phase change material partially melts owing to Joule heating, causing the crystallization of part of the amorphous area. Continuous conductivity levels can be achieved by controlling the amplitude, duration, and quantity of enhancement pulses to control the degree of crystallization. Based on this, this synaptic device can perform various synaptic functions, such as spike-timing-dependent plasticity (STDP) and long-term potentiation (LTP). Another source of Joule is that the phase change material is connected to the heating element, allowing heating to occur in the area near the heater. Sung et al. fabricated a threshold switch-phase-change memory consists of an Ag-doped SiO_2_ threshold switch and GST-based phase-change memory (Fig. 7c) [221]. Under the action of electric field, a volatile conductive filament grows in the Ag-doped SiO_2_ threshold switch layer and forms contact with the GST/SiO_2_ interface. The phase change of the top GST film is induced by Joule heating of conductive filament because of the small contact area of the conductive wire. Compare to heating inside the phase change material, the devices used a conductive filament as a heater, which can obtain low-power phase transition, excellent endurance and attained large resistance ratio. GST-based devices exploit the phase-change mechanism to achieve multimodal sensing by modulating phase transition temperatures or generating heat for indirect detection of optical and chemical signals [222, 223]. When GST thin films absorb photons of sufficient energy, photo-thermal heating elevates local temperatures above the melting point and subsequent rapid quenching transforms the crystalline phase into a high-resistance amorphous state [206]. Furthermore, UV irradiation reduces phase transition temperatures and alters phase-change kinetics, enabling distinct optical signal detection pathways [224]. For chemical sensing, adsorption of specific gases (e.g., NH_3_, hydrogen sulfide (H_2_S)) on GST surfaces modifies crystallization kinetics through surface dipole formation or charge transfer, thereby lowering or raising crystallization threshold temperatures to induce or inhibit phase transitions [225].

Apart from the conventional phase change transforming between amorphous and crystalline phases, there exists a unique structural phase transition mechanism. This mechanism enables a system to transition from one steady phase to another under various external stimulations, such as electric field, ion migration, pressure, and temperature, ultimately leading to a change in conductance [206, 226, 227]. The conventional phase transition materials are the transition metal dichalcogenides (TMDs) [228, 229]. TMDs generally possess several different stable phases, including 2H, 1T, 1T′, Td and 3R phases [230]. Usually, TMD materials exhibit structural phase transitions between the distorted octahedral structure (1T or 1T′ phase) and the trigonal prismatic structure (2H phase) [227, 231, 232]. Memristors based on phase transition mechanism have been widely used as artificial synaptic devices, which have advantages in scalability, durability, reliability and multi-level programming resistance [233, 234]. Compared with the amorphous to crystalline transitions in PCM, memristors based on phase transition mechanisms have high capabilities for realizing reliable and fast switching multi-level states [227, 231].

The phase transition mechanism can be realized under various external stimulations, such as electrical, optical, pressure and temperature signals [138, 231, 235, 236]. Li et al. fabricated a based-vanadium dioxide (VO_2_) integrated neuromorphic sensor array (Fig. 7d) to sense optical signals [224]. Optically induced oxygen vacancies can cause electronic phase transitions. The tuning of phase transition can be achieved by controlling the intensity and persistence of ultraviolet illumination. Based on the reversible regulation of VO_2_ films by ultraviolet illumination, the neuromorphic ultraviolet sensor can simultaneously achieve sensing, memory, and processing functions. In addition to the above stimulation, there are also some factors that can cause structural phase transitions, such as material thickness, temperature, etc. Yu et al. designed a graphene-assisted non-volatile phase transition strategy for artificial optoelectronic synapses based on VO_2_ nanoparticle/graphene heterojunction (Fig. 7e) [237]. VO_2_ with photo-induced phase transition properties forms a heterojunction with graphene. Graphene helps to achieve non-volatile phase transitions of VO_2_ and amplifies the signals generated by the phase transition. By applying external optical or electrical stimuli to modulate the gate voltage on graphene Fermi level and regulate electron flow between VO_2_ and graphene, the electronic concentration in VO_2_ is altered, thereby inducing phase transitions. This enables reversible and stable synaptic conductance modulation. Yuan et al. realized a memristor based on epitaxial VO_2_ and a neuromorphic sensing system composed of calibratable artificial sensory neurons based on epitaxial VO_2_ (Fig. 7f) [238]. Artificial sensory neurons can be utilized to construct various spiking sensory neurons capable of sensing physical signals and converting them into spikes. By adjusting the resistance range of diverse sensors to desired states through scaling resistors, these neurons can adapt to multiple sensor types. Based on this, a multimodal perceptual system capable of encoding pressure, curvature, temperature, and optical signals into electrical spikes is demonstrated by integrating artificial sensory neurons with pressure, curvature, optical, and temperature sensors (Fig. 7g). The core of achieving simultaneous multi-stimulus perception and fusion in phase transition-based multimodal sensing devices lies in mapping diverse physical quantities (electrical, optical, pressure, temperature) onto a unified phase transition order parameter (e.g., V–V dimer distance in VO_2_) [239]. Through cooperative regulation of the phase transition free-energy barrier by external fields, the device’s conductance becomes a continuous multivariable function. This response intrinsically originates from material-specific nonlinear coupling, enabling physical information fusion without external conversion [240]. Concurrently, significant temporal separation exists in response dynamics across stimuli (optical: fs-scale, electrical: ns-scale, pressure/temperature: μs-ms scale). This allows signal decoupling and feature extraction at the device/circuit level, ultimately achieving integrated perception-fusion-decision functionality.

The phase-change mechanism primarily exploits reversible crystalline–amorphous transitions, predominantly driven by Joule heating. Here, resistance switching originates from bandgap changes induced by atomic rearrangement, thereby enabling non-volatile electrical signal storage [214]. However, its stimulus detection capabilities are generally confined to thermal, optical, and electrical inputs. Memristors based on phase change require external algorithms to distinguish different stimuli and depend on auxiliary circuitry to convert multimodal signals into electrical signals [164]. In contrast, the phase transition mechanism involves electronic/structural phase transformations activated by multiple physical fields: electric fields modify orbital occupancy while photons excite lattice vibrations [241, 242]. This enables single devices to natively respond to optical, thermal, pressure, and electrical signals, facilitating volatile conductivity modulation that intrinsically achieves multimodal fusion without supporting circuits. Crucially, identical lattice parameters in phase transition memristors exhibit distinct sensitivities to different stimuli, enabling direct signal decoupling at the device level [243].

Phase-change materials possess multi-stimuli responsiveness and state uniformity [244–246]. The phase transition thresholds of these materials (e.g., temperature, electric field intensity, and light intensity) can be modulated through material design, enabling diverse input signals (optical, electrical, and temperature) to trigger phase transitions by supplying energy [207, 246]. Additionally, the threshold characteristics of phase transitions and dynamic cumulative effects (e.g., repeated weak signals inducing phase transitions) closely align with the information processing mechanisms of biological neurons [246, 247]. By engineering materials (e.g., heterostructures and doping), phase transition thresholds can be tailored to adapt to multiple signals, achieving multi-parameter collaborative regulation [248].

The primary reason why neuromorphic devices enable multimodal perception via phase transition mechanisms lies in the inherent capability of phase-change materials. This capability can unify the energy from diverse physical signals (light, electricity, and temperature) into nonlinear transitions of internal phase states, which are directly output through phase-dependent electrical properties (e.g., resistance) [40, 249]. Multimodal perception via phase transition mechanisms is achieved through the pronounced resistance and optical property changes induced by reversible crystalline–amorphous phase transitions, enabling the detection and discrimination of distinct signal types [250, 251]. Moreover, these devices leverage inherent differences in the energy thresholds, temporal scales, or pulse shapes required to trigger phase transitions across different modalities for signal differentiation [252, 253]. This “multi-input, single-state, single-output” mechanism not only overcomes the limitations of traditional sensors' discrete designs but also physically emulates the multisensory information integration ability of biological neurons [254, 255]. It thereby provides a core material foundation for constructing efficient, compact, brain-inspired sensing systems.

### Electrochemical Doping

**Fig. 8:**
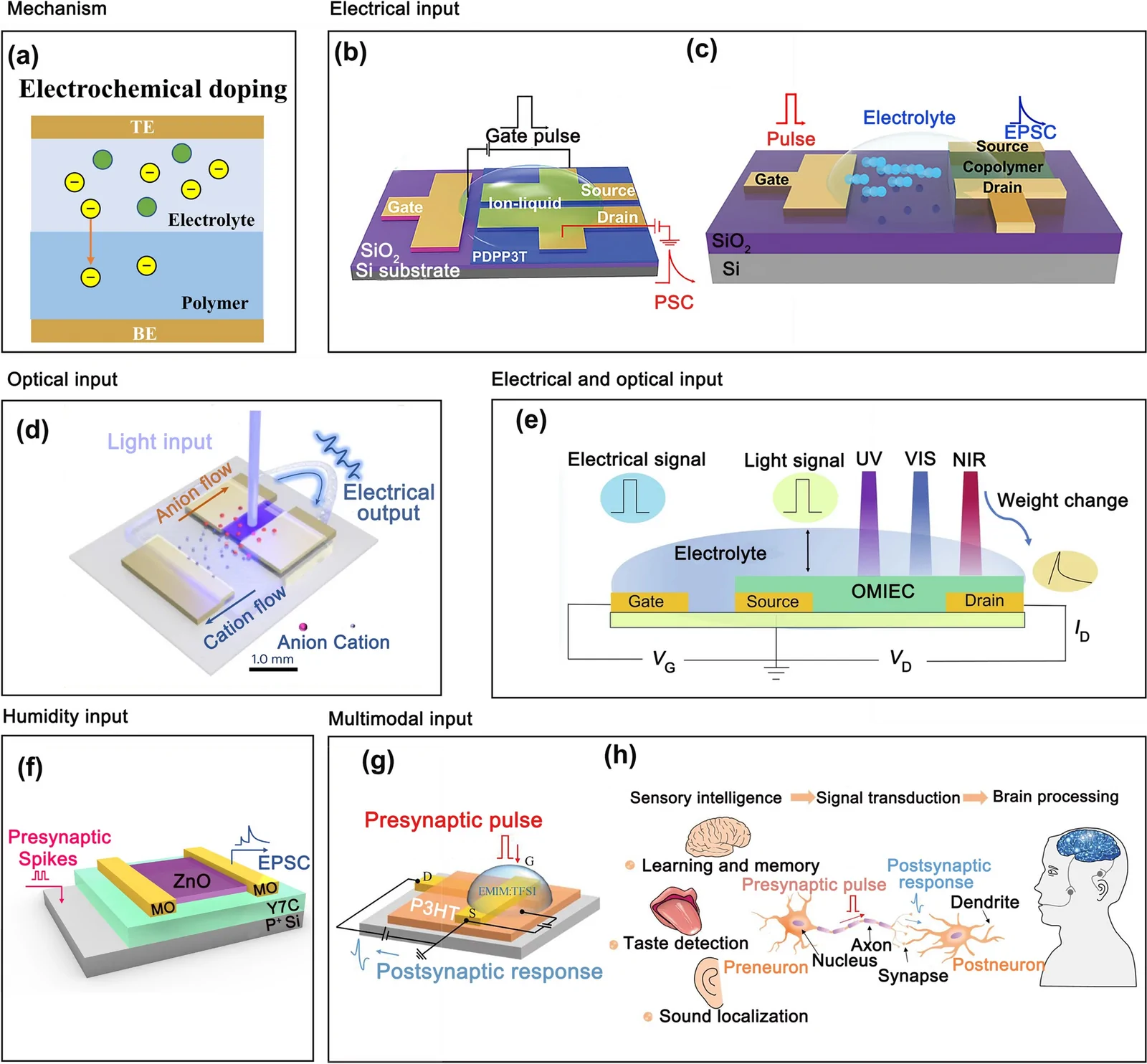
Electrochemical doping synaptic devices processing multimodal stimuli. **a** Resistive switching mechanism via electrochemical doping. **b** OECT biomimetic synapse with electrical input. Reproduced with permission [266]. Copyright 2024, Advanced Functional Materials. **c** Random copolymer transistor schematic with electrical input. Reproduced with permission [267]. Copyright 2025, Small Structures. **d** Organic optoelectronic synapse with optical input. Reproduced with permission [268]. Copyright 2023, Nature Photonics. **e** Optoelectrochemical *n*-type OECT with optical/electrical inputs. Reproduced with permission [269]. Copyright 2025, Nature Communications. **f** ZnO/SiO_2_ transistor with humidity input. Reproduced with permission [270]. Copyright 2022, Journal of Materials Science & Technology. **g** Ionic gate synaptic transistor. Reproduced with permission [271]. Copyright 2022, Advanced Functional Materials.** h** Biological sensory signal processing with multimodal stimuli of **g**

The electrochemical doping mechanism is a core technology that enables reversible switching of resistance states by dynamically modulating carrier concentration through ion insertion/extraction or redox reactions in materials (Fig. 8a) [256, 257]. This mechanism can mimic the long-term plasticity of biological synapses, providing a physical foundation for constructing low-power, high-density neuromorphic computing systems. In neuromorphic devices, the electrochemical doping mechanism is widely employed to achieve resistance switching [258]. Its core lies in charge transfer and chemical potential shifts, inherently dependent on interfacial reactions between electrodes and electrolytes. Under applied voltage or current, these reactions trigger doping/de-doping processes to modulate the resistive states of the device [259, 260]. Active materials for this mechanism primarily include conductive polymers (poly(3,4-ethylenedioxythiophene):poly(styrene sulfonate) (PEDOT: PSS)), transition metal oxides (tungsten trioxide (WO_3_), MoO_3_), and two-dimensional materials (MXene) [128, 261, 262]. Both ion migration and electrochemical doping mechanisms utilize ionic kinetics. Table 1 compares their distinctions across five key aspects, thus enabling direct comparison of their operating principles.

**Table 1:** Ionic migration versus electrochemical doping

Parameter	Ionic migration	Electrochemical doping
Primary Mechanism	Field-driven ion drift	Redox-driven ion intercalation
Reversibility	Partially reversible	Highly reversible
Carrier Change	Conductive filament morphology	Bulk carrier density
Material Requirement	Ionic conductivity	Redox activity
Representative System	Ag/GeS_2_/Pt (Ag^+^ migration)	Li/LiPON/WO_3_ (Li^+^ doping)

The electrochemical doping mechanism relies on electric field-driven ion migration within active materials and the resulting charge compensation effects [263]. When voltage is applied, ions in the electrolyte (e.g., H⁺, Li⁺, and Cl⁻) migrate to the active layer (e.g., conductive polymers and transition metal oxides) under the electric field, inducing oxidation or reduction reactions that modify doping level of the material [264]. Under forward bias, ion injection increases carrier concentration, lowering resistance; under reverse bias, ion extraction reduces carrier concentration, increasing resistance [262]. Architectures based on the electrochemical doping mechanism offer a large conductance modulation window and are fully compatible with flexible substrates [265]. Nevertheless, ion relaxation delays responses, and electrolyte leakage risks impair long-term reliability [71].

The electrochemical doping mechanism of neuromorphic devices can be driven by electrical signals. Electrical signals directly drive the migration of ions at the electrolyte/material interface, thereby regulating ion intercalation/deintercalation. Lee et al. fabricated a polymer-based memristor, which is modulated by the electrochemical doping mechanism driven by an electric field (Fig. 8b) [266]. A strategy was proposed to enhance electrochemical doping and de-doping by utilizing different coulombic ions. The research results indicate that doping ions in the channel layer affect inter-ion interactions, and influencing the non-volatile effect by improving the doping performance of the synaptic device. When a pulse is applied to an electrolyte, anions form an EDL at the electrolyte/channel interface. However, following the pulse application, the accumulated anions rapidly dissipate from polymer, leading to a swift back-diffusion into the electrolyte. This indicates that EDL depolarization has occurred, resulting in the restoration of the electrochemical potential at the solid–liquid interface to its initial state. Sung et al. fabricated electrolyte-gated organic synaptic transistor structures through copolymerization between two different polymers, which induced superior non-volatility through more effective electrochemical doping via ions (Fig. 8c) [267]. By utilizing anion movement generated by electrical pulses to drive EPSC that mimic neural transmission, artificial synapses have achieved.

In addition to electrical signals, optical and humidity signals can also regulate the electrochemical doping mechanism of neuromorphic devices. Chen et al. reported an organic optoelectronic synapse realized through photon-modulated electrochemical doping in an electrochemical transistor (Fig. 8d) [268]. In the synaptic device, optical signal can facilitate the insertion of ions into a photoactive layer composed of a donor–acceptor heterojunction interface. This approach enables high-density multi-level conductance modulation and emulation of synaptic activities inherent to biological systems through ion flux manipulation. The light absorption in donor–acceptor heterojunctions enables photogeneration of charge carriers, which perturbs electrochemical doping while facilitating anion migration from the electrolyte for charge compensation in the channel. Consequently, the elevated carrier concentration generated via photon-modulated doping manifests as increased drain current. Upon light cessation, residual anions surrounding the doped polymer matrix prevent immediate charge recombination, inducing gradual current decay that contributes to non-volatile memory retention. By employing a single-component organic mixed ionic-electronic conductor as the channel in OECTs, wang et al. developed an ionically gated optoelectrochemical synapse (Fig. 8e) [269]. The device demonstrates dual responsiveness to optical and electrical stimuli delivered via aqueous electrolytes, enabling neuromorphic modulation through synergistic ionic-electronic interactions. Under illumination, the channel becomes more negatively polarized, which electrostatically attracts additional cations. Under combined optical-electrical biasing, a film mass increases attributable to cation intercalation—a photonic control mechanism analogous to the effect of elevated voltage application, consequently inducing an enhanced doping state. Subsequent to optical signal removal, electronic recombination processes may occur while excess cations gradually egress from the polymer matrix, resulting in a gradual nonlinear decay of channel current that manifests in charge retention characteristics. Song et al. presented ZnO-based artificial synapses with peptide insulators for the electrical emulation of biological synapses, which can be affected by humidity signal (Fig. 8f) [270]. The dielectric constant of peptide membranes exhibits humidity-dependent enhancement due to the formation of protonic EDL. Under low-humidity conditions, proton transport remains inhibited at small gate voltages, resulting in ineffective electrostatic gating. Conversely, elevated humidity triggers significant proton mobility within the hydrated film, where proton-dominated gating mechanisms prevail. This ionic dynamic leads to excitatory EPSC with retarded decay kinetics, demonstrating non-volatile memory behavior through proton redistribution hysteresis. As discussed above, neuromorphic devices can detect optical and humidity signals through electrochemical doping mechanisms. For optical signals, photogenerated carriers directly drive ion migration, facilitating electrochemical doping. For humidity signals, humidity adsorption alters the ion concentration on the material surface.

Simultaneously, multimodal recognition can be achieved through electrochemical doping mechanism. Liu et al. engineered a polymer-based electrolyte-gated vertical organic field-effect transistor architecture, demonstrating neuromorphic artificial synapses with multisensory integration capabilities (Fig. 8g) [271]. This device platform enables biomimetic emulation of human cross-modal perception, particularly gustatory-auditory sensory fusion, through ion-modulated adaptive signal transduction and stimuli-responsive synaptic plasticity (Fig. 8h). Inspired by human taste perception, an artificial tongue was designed to detect acidity. An ionic liquid serves as a thin saliva-like layer on the tongue. Various acidity levels were achieved by injecting acetic acid into the ionic liquid. Dropping different acetic acid concentrations onto the taste sensor array caused distinct current changes at specific points, generating taste mapping. This demonstrates simultaneous detection capability of the fabricated tongue for varying acidity levels.

Electrochemical doping offers several unique advantages, as it enables direct coupling with multiple energy forms through ion migration [111]. Moreover, electrochemical doping exhibits self-adaptive reversibility, where the doping process can be restored to its initial state via reverse electric fields or ion diffusion [1]. Additionally, it demonstrates intrinsic sensitivity to chemical environmental changes (e.g., gases, humidity, pH) through direct signal conversion via ion–molecule interactions [272]. The primary reason why neuromorphic devices achieve multimodal sensing through electrochemical doping mechanisms lies in the deep integration of two key functionalities. This integration combines the intrinsic unified conversion of diverse physical/chemical signals with the biologically level dynamic response enabled by ion migration-based dynamic regulation [94, 273]. This mechanism not only demonstrates universality in energy conversion forms but also exhibits high compatibility with the ion channel behaviors of biological neurons in terms of bionic characteristics.

### Synaptic Mechanisms for Multimodal Integration

In neuromorphic systems designed for multimodal integration, the efficacy of synaptic mechanisms hinges on their ability to reconcile diverse signal characteristics while maintaining biological plausibility [274, 275]. Multimodal integration necessitates synaptic platforms with broad dynamic ranges to accommodate amplitude disparities across sensory modalities, linear tunability for precise cross-modal weight allocation, temporal consistency to synchronize heterogeneous response timescales (milliseconds to minutes), and ultralow power consumption compatible with edge computing constraints [276–280]. Environmental robustness against temperature and humidity fluctuations that disrupt multimodal signals is a critical requirement for practical deployment [281].

The suitability of various synaptic mechanisms differs significantly across input signals and multimodal integration scenarios [177, 282, 283]. Ferroelectricity mechanism, based on polarization reversal in ferroelectric materials, offer advantages such as offer non-volatile memory and low power consumption, yet their slower switching speeds and nonlinear weight updates may compromise precise multimodal signal modulation [284, 285]. Charge trapping mechanism modulates conductivity through reversible charge trapping/de-trapping, but their limited charge retention and narrow dynamic range render them suboptimal for multimodal applications requiring long-term stability [286]. Phase change materials exhibit high on/off ratios and stability through crystalline–amorphous transitions, but their high energy consumption and sluggish phase transition kinetics hinder flexibility in processing rapidly varying signals [287]. The ion migration mechanism alters material conductivity through ionic movement, featuring rapid response and a wide dynamic range [137]. However, its long-term reliability requires verification due to potential instability issues associated with ion migration processes [142]. Electrochemical doping mechanisms employ electrochemical reactions to adjust material doping levels, demonstrating superior reversibility and dynamic modulation capabilities that render them suitable for emulating the continuous plasticity of biological synapses [268]. However, their implementation may necessitate complex electrolyte environments and pose integration challenges due to system complexity [262]. Conductive filament mechanism achieves a high on/off ratio and fast switching through filament formation/rupture, but their discrete switching behavior and poor linearity limit precise analog-like synaptic weight tuning [127]. Each mechanism presents distinct trade-offs in balancing speed, linearity, stability, and integration feasibility for multimodal neuromorphic systems.

In summary, electrochemical doping and ion migration mechanisms demonstrate superior suitability for multimodal signal fusion. Ion migration exhibits high linearity and continuous tunability, enabling smooth weight updates through electric field-regulated ion redistribution, which proves ideal for fine-grained multimodal integration. Its rapid response and ultralow power consumption meet the real-time demands of multimodal systems. Crucially, ion migration achieves direct cross-modal coupling by translating physical signals (e.g., pressure and temperature) into ionic mobility variations without requiring external conversion modules. For instance, flexible ionogel sensors transduce mechanical strain into ion transport path modulation, enabling concurrent pressure–temperature perception. Electrochemical doping offers an ultrahigh dynamic range, accommodating multimodal amplitude disparities through ion intercalation/deintercalation-driven doping adjustments. WO_3_-based electrochemical transistors, for example, achieve linear responses across optical and mechanical stimuli. This mechanism inherently couples chemical, optical, electrical, and pressure signals, with heightened sensitivity to environmental variations (e.g., gas concentration and humidity), making it particularly suitable for complex multimodal integration. The synergistic combination of ion migration and electrochemical doping in heterostructures allows rapid fine-tuning via ion migration while leveraging electrochemical doping for large-span signal processing. Owing to their exceptional linearity, dynamic range, and direct signal transduction capabilities, these mechanisms emerge as optimal choices for multimodal integration. Ion migration excels in scenarios requiring rapid, continuous modulation (e.g., tactile-visual synchronization), whereas electrochemical doping dominates in cross-physical-field coupling (e.g., chemical–thermal–optical fusion). Ferroelectricity and charge trapping mechanisms may serve supplementary roles to enhance system robustness. Future advancements in heterostructure design and interfacial engineering are expected to solidify their dominance in multimodal neuromorphic systems.

## Multimodal Signal Data Fusion

### Single-Modality and Memristor-Based Multimodal Systems

Neuromorphic systems relying on single-modality signal processing for decision-making often face inherent uncertainties due to signal stochasticity, incompleteness, and noise amplification during information extraction [288, 289]. The absence of cross-modal validation mechanisms further limits their perceptual robustness. A direct solution to the problems of insufficient environmental adaptability and missing information redundancy caused by single-signal processing capabilities is the implementation of multimodal sensory fusion [290]. Multimodal neuromorphic systems enable multidimensional data integration, simultaneously processing visual, tactile, auditory, and other signals through complementary information to mitigate the limitations of individual modalities [274]. These systems exhibit enhanced environmental adaptability, maintaining stable perception in complex scenarios (e.g., low-light or high-noise environments) by leveraging redundant data [291]. Moreover, their superior robustness and fault tolerance ensure operational continuity through alternative sensory inputs when specific modalities become compromised [292].

Multimodal sensory fusion directly addresses the limitations of single-signal processing systems by enabling multidimensional data integration. Traditional single-modality architectures, limited to processing isolated signal types (e.g., pure visual or thermal data), rely on post-hoc software fusion of multisource inputs, leading to information loss and computational latency [293]. In contrast, multisensory neuromorphic systems achieve hardware-level integration of visual, tactile, auditory, and other sensory streams through complementary information processing, mitigating individual modal constraints [274, 294]. Single-modality systems require repeated activation of redundant neurons to process complex scenarios and suffer from high interface latency in central processing units [295]. In contrast, multimodal neuromorphic systems employ event-driven computation and distributed processing, activating memristors only when multi-sensors are synergistically triggered, thereby significantly reducing static power consumption [296]. In dynamic environments (e.g., abrupt illumination changes), single-modality systems exhibit severe performance degradation (e.g., huge recognition accuracy drop in pure vision systems), while multimodal neuromorphic frameworks enhance robustness through cross-modal suppression mechanisms [297, 298]. Additionally, the non-volatile characteristics of memristors enable real-time synaptic weight updates, facilitating adaptive calibration to environmental variations (e.g., temperature drift) and improving overall adaptability [299]. This architecture thus provides a neuromorphic-specific solution for real-time, energy-constrained applications by merging sensory diversity with hardware-efficient computation.

Compared to traditional single-modality sensing, directly integrating unimodal sensors with memristors to form sensing-memory-computing integrated units offers significant advantages, which stem from the unique physical properties and in-memory computing capabilities of memristor. Traditional single-modality sensing systems, such as vision only or tactile only configurations, exhibit limitations in complex dynamic environments. In contrast, memristor-integrated systems leverage non-volatile properties to achieve environmental self-adaptation. For instance, photomemristors in visual systems implement dynamic threshold modulation to maintain stable performance across varying illumination conditions [300]. Traditional unimodal sensing systems typically require analog-to-digital conversion during signal processing, a procedure that increases system complexity and risks information loss [20]. In contrast, memristors inherently process continuous signals, as demonstrated by the continuous pressure-to-resistance mapping in tactile sensors [40]. This eliminates signal conversion requirements while enhancing system processing efficiency and accuracy. Whereas traditional systems rely on backend algorithms for data processing and learning, incurring high power consumption and latency [295]. Memristor crossbars enable edge online learning, as demonstrated by Tsinghua University’s monolithic integrated memristor chip that supports on-chip learning with merely 3% energy consumption of conventional application-specific integrated circuits (ASICs) [301]. This hardware-level learning capability concurrently facilitates dynamic parameter tuning and optimization during real-time data processing. Furthermore, traditional single-modality sensing systems require discrete sensor, analog-to-digital converter (ADC), and processor chips with interconnects, resulting in large footprints and system complexity. In contrast, memristors adopt crossbar architectures that enable ultrahigh-density integration [302].

The fundamental rationale for integrating single-modality sensing with memristors rather than other two-terminal devices lies in the memristor's unique capacity for sensing-memory-processing convergence. This eliminates data shuttling, sidesteps analog-to-digital conversion and delivers edge intelligence [303]. Whereas alternative two-terminal components deliver only singular functionalities. Memristors provide intrinsic non-volatility that preserves resistive states without external power, enabling environmental self-adaptation in unimodal systems [304]. In contrast, traditional two-terminal devices such as resistors and diodes lack this persistent state retention capability, making them inadequate for complex environmental variations. Memristors support continuous resistance modulation through multi-state switching under electrical stimuli, enabling direct processing of analog signals to enhance efficiency and accuracy [305]. In comparison, traditional resistors exhibit fixed resistance values, diodes enable only unidirectional current flow, and while transistors provide switching capabilities, they lack continuous modulation capacities. These inherent limitations collectively prevent conventional two-terminal devices from meeting the analog signal processing requirements of single-modality systems. In terms of energy efficiency, memristors exhibit exceptional energy efficiency performance, with power consumption significantly lower than conventional transistors [152]. This advantage becomes particularly pronounced in neural network processing tasks, where energy efficiency improvements can span multiple orders of magnitude. Meanwhile, traditional two-terminal devices like transistors face scaling mismatch challenges due to their charge-based mechanisms and require substantial programming currents, leading to increased overall power consumption [306]. Furthermore, memristors possess hardware-level learning capabilities by emulating synaptic weights through conductance values, enabling real-time updates of electrical properties [307]. This allows single-modality systems to achieve online learning and dynamic parameter adjustment via memristor arrays. In contrast, traditional two-terminal devices lack inherent learning mechanisms and must rely on backend algorithms for such functionalities, which not only increases system complexity but also constrains real-time performance [308]. Compared to memristors, other two-terminal devices exhibit critical limitations. Resistors have fixed resistance values incapable of environmental adaptive calibration. Capacitors suffer from charge leakage requiring refresh circuits that increase power consumption and system complexity. Diodes only possess binary switching characteristics unable to represent continuous sensor signals. Traditional resistive random access memories (RRAMs) are limited to binary storage, thereby losing analog computing capabilities.

### Single-Memristor Multimodal Sensing System

Conventional systems, which rely on multiple discrete sensors and dedicated processing units, suffer from high hardware complexity, bulky form factors, and significant power consumption [295, 309]. The signal conversion between sensors necessitates additional ADCs and interface circuits, leading to substantial energy loss [295]. Multimodal signals require post-processing fusion via central processing units (CPUs)/graphics processing units (GPUs)), introducing millisecond-level latency that struggles to meet real-time requirements [295]. Furthermore, the serialized processing paradigm of discrete architectures results in computational inefficiency and limited parallelism. Physical separation of multiple sensors also risks signal interference, while environmental fluctuations (e.g., temperature and humidity) induce sensor drift discrepancies, demanding complex calibration algorithms [310]. To address these challenges, a single-memristor multimodal sensing system offers an effective solution. Single-memristor multimodal systems eliminate the need for independent sensors by directly leveraging the multi-physical response characteristics of memristive materials. The primary advantage lies in their ultra-simplified hardware architecture and exceptional integration density [311]. Through heterogeneous structural design, these systems demonstrate ultra-wide dynamic range capabilities, enabling simultaneous detection of diverse signals with varying amplitude ranges [312]. Reconfigurable modal weights can be implemented via mechanism design, such as electric field or optical modulation of ionic migration pathways, dynamically prioritizing dominant sensing modalities [313]. By directly modulating memristive conductance states through input signals, the system achieves low-latency operation and ultralow power consumption [314].

**Fig. 9:**
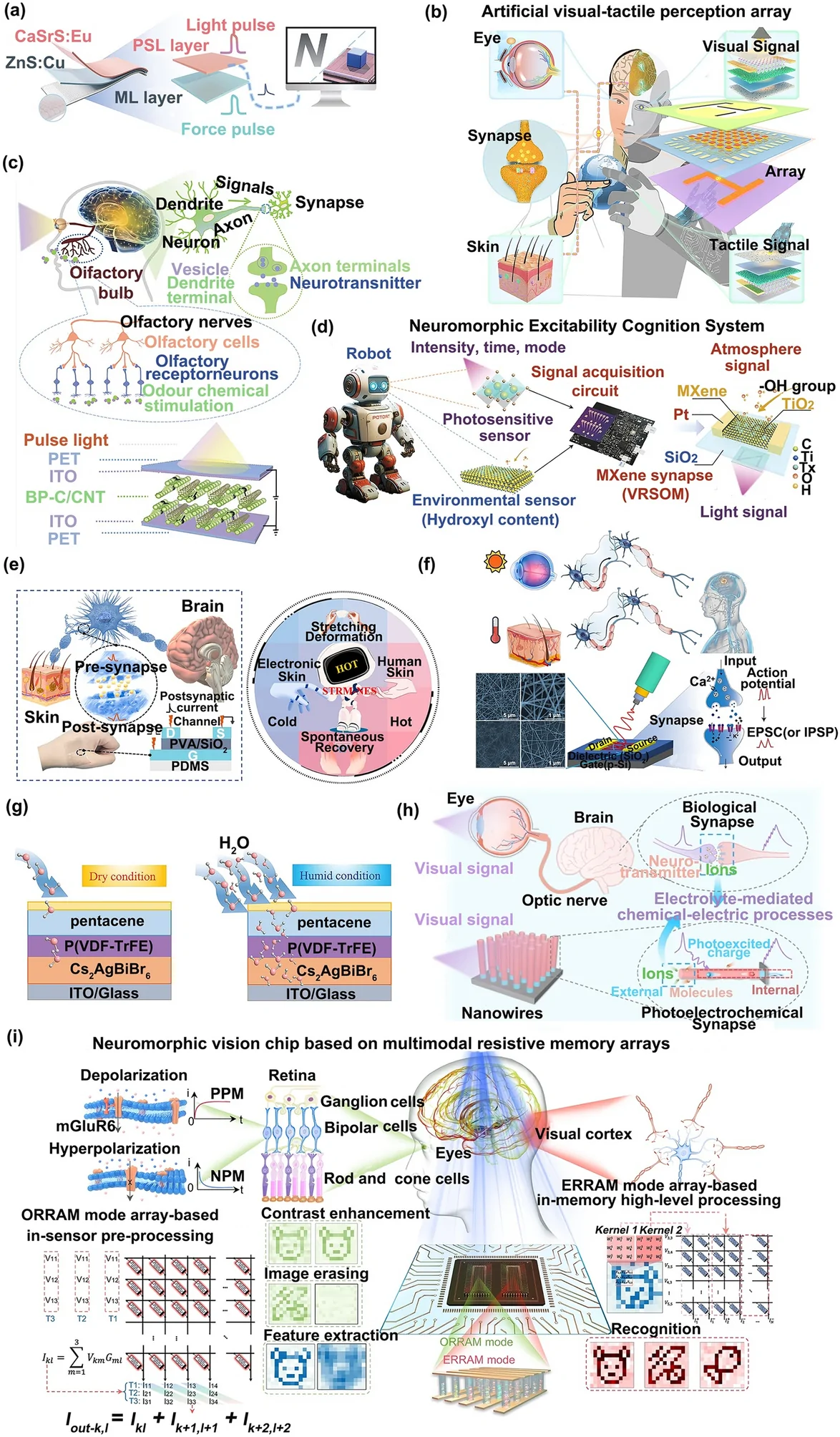
Internal multisensory perception integrated with memristors. **a** Visual-tactile synapse with mechanoluminescent/photo-stimulated layers. Reproduced with permission [315]. Copyright 2025, Advanced Materials. **b** Visual-tactile perception array combining mechanoluminescent layer and photoelectronic synapse network. Reproduced with permission [316]. Copyright 2023, InfoMat. **c** Visual-olfactory multisensory integration device. Reproduced with permission [317]. Copyright 2024, Advanced Science. **d** Bio-inspired visual-respiratory cognitive nerve with arrayed circuits. Reproduced with permission [318]. Copyright 2024, Advanced Materials. **e** Stretchable temperature-responsive e-skin with multimodal synaptic transmission. Reproduced with permission [319]. Copyright 2022, ACS Nano. **f** InMgO NFs synaptic device for visual/temperature perception. Reproduced with permission [320]. Copyright 2024, International Journal of Extreme Manufacturing. **g** Humidity-modulated neuromorphic behavior mechanism. Reproduced with permission [321]. Copyright 2023, Advanced Materials Technologies. **h** Biological visual system and photoelectrochemical synapse. Reproduced with permission [322]. Copyright 2024, Nature Communications. **i** Multimodal memristive array vision chip. Reproduced with permission [10]. Copyright 2023, Nature Communications

A common method for implementing multimodal sensing in single-memristor sensing systems involves designing functional layer architectures. Mechanoluminescence (ML) allows for the quantitative conversion of mechanical stimuli into light emission in a real-time and in situ manner. This force-to-light conversion enables the construction of visual-tactile sensors without the need for electrical or optical power sources. Guo et al. developed an artificial visual-tactile synapse for in-sensor computing enabled by the consisting of photo-stimulated luminescence (PSL) material and mechanoluminescent layer (Fig. 9a) [315]. The artificial synapse consists of three layers. The first layer is composed of PSL phosphor with photon-capturing capability, followed by a layer of ML material that emits light driven by mechanical force. The bottom layer, known as the mechanical microstructure layer, enhances the mechanical sensitivity of the device. The ML layer and microstructure collaboratively convert mechanical signals into optical outputs to modulate synaptic plasticity. Mechanical signals are directly transduced into light emission via ML materials, which optically stimulate the adjacent PSL layer without requiring pre/post-illumination. The PSL layer operates as both photon reservoir and in-memory computing unit, leveraging its photon-trapping capacity and electron de-trapping processes under near-infrared (NIR) irradiation. Mechanical forces (presynaptic input 1) and visible light (presynaptic input 2) serve as analog stimuli, while PSL optical signals function as postsynaptic responses. He et al. proposed an artificial visual-tactile perception array consisting of an integrated mechanoluminescent layer and a photoelectronic synapse network (Fig. 9b) [316]. The compact device integrates an IGZO/methylammonium lead iodide (MAPbI_3_) heterostructure and a ML layer. The IGZO/MAPbI_3_ heterostructure serves as the underlying layer for visual sensing and artificial synapses, while the ML layer transduces mechanical stimuli into light for tactile sensing and synaptic plasticity modulation. This bimodal modulation of visual-tactile stimuli enables enhanced processing, learning, recognition, and memorization of stimulus information. Dong et al. fabricated a bionic photo-olfactory multisensory artificial synapse device using a two-dimensional/one-dimensional (2D/1D) black phosphorus–carbon–carbon nanotube (BP-C/CNT) heterostructured filter membrane as the functional active layer (Fig. 9c) [317]. By simultaneously integrating optical modulation, gas sensing, and synaptic functionalities within a single device, this system emulates the characteristics and operational capabilities of biological multisensory neurons. The polyethylene terephthalate (PET)/ITO top electrode corresponds to the presynaptic membrane, while the bottom ITO/PET layer functions as the postsynaptic membrane, with the BP-C/CNT functional layer serving as the synaptic transmission medium. The PET/ITO electrode acts as the presynaptic membrane to receive stimulus signals, where electrons are injected from the PET/ITO side, simulating neurotransmitter release. These electrons traverse the BP-C/CNT layer before reaching the graphite bottom electrode, generating a synapse-mimetic current interpreted as postsynaptic current. Gas molecules readily adsorb onto the BP-C/CNT surface, undergoing dehydrogenation reactions with oxygen anions at the material interface. This process releases electrons, altering surface potential barriers and modulating internal electron concentration. Under optical excitation in gaseous environments, the photoelectric effect in BP-C/CNT generates abundant photogenerated carriers. These carriers are partially captured by surface traps, replenishing internal charges and further modifying electron concentration. Gas adsorption and photogenerated carriers synergistically modulate electronic states, emulating bio-neural co-regulatory mechanisms.

Unlike the elaborately designed architectures of multisensory neuromorphic devices mentioned above, certain implementations achieve multimodal synergy through a single material. Tan et al. introduce a bio-inspired multisensory integrated cognitive nerve consisting of the an artificial visual-respiratory synapse and corresponding arrayed reading circuits (Fig. 9d) [318]. In the designed visual-respiratory synapse system, monolayer oxidized MXene nanosheets enable bimodal sensing of optical and airflow signals. Visual signals are emulated via photocurrent generation through titanium dioxide (TiO_2_) crystals, while airflow stimuli induce hydroxyl-oxygen vacancy interactions to mimic respiratory-arousal-modulated relaxation behaviors, analogous to biological ocular-nasal systems. Visual and respiratory modalities are synergistically activated by externally controlling light source intensity/duration to reconstruct naturalistic visual scenes. This multisensory integration achieves event-based synaptic signal readout in real-time through output load circuitry. Wang et al. developed a stretchable temperature-responsive multimodal neuromorphic electrical skin that integrates temperature sensing, mechanical perception, and synaptic functionalities (Fig. 9e) [319]. The device employs a polyvinyl alcohol (PVA)/SiO_2_ stacked structure as the gate dielectric. The abundance of hydrogen bonds in the PVA hydrogel constitutes the primary rationale for its application in neuromorphic synaptic devices. Elevated temperatures alter hydrogen-bond interactions within the hydrogel, increasing intermolecular distances and thereby enhancing proton hopping probability in PVA for synaptic plasticity modulation. This architecture enables concurrent pressure–temperature perception through PVA-mediated mechanisms, achieving synergistic multimodal signal integration. Wen et al. proposed a indium-magnesium oxide (InMgO) nanofibers (NFs) synaptic device with visual and temperature perception (Fig. 9f) [320]. The InMgO material is rich in oxygen vacancies. When light irradiates the InMgO nanochannels of the device, the oxygen vacancies undergo ionization to generate free electrons and VO^2+^ charge centers. The temperature sensing mechanism of InMgO originates from its thermally activated carrier characteristics and the ionization activation energy that decreases with rising temperature. The lower ionization activation energy facilitates the excitation of a higher density of photogenerated carriers under the same light intensity. Based on this principle, the device achieves collaborative perception and integration of photo-thermal dual-modal signals through InMgO. Lao et al. designed self-powered two-terminal optoelectronic synapse based on a lead-free cesium silver bismuth bromide (Cs_2_AgBiBr_6_)/P(VDF-TrFE)/pentacene heterostructure, which shows bidirectional responses to optical signal and humidity signal (Fig. 9g) [321]. Cs_2_AgBiBr_6_ exhibits high humidity selectivity, strong light absorption, and efficient photoelectric conversion, rendering it sensitive to both humidity and optical signals. This enables the modulation synaptic performance of the device through the synergistic integration of humidity and light pulses. Liu et al. reported an optoelectronic synaptic device based on semiconductor nanowires composed of p-type aluminum gallium nitride (p-AlGaN)/n-type gallium nitride (n-GaN) heterostructures, which demonstrates dual sensing capabilities for both chemical and optical signals (Fig. 9h) [322]. GaN material exhibits excellent optoelectronic properties and demonstrates good chemical stability in electrolyte solutions, making it suitable for studies involving electrolyte-mediated chemical reactions and ideal for constructing optoelectronic synapses with chemically relevant functionalities. Synaptic responses can be modulated by either chemical modifications on the nanowire surfaces or alterations in the external electrolyte environment. Upon illumination, charge carriers are generated within the nanowires. A portion of these carriers accumulate within the nanowires, inducing optoelectronic synaptic responses. Meanwhile, ions and molecules in the electrolyte consume another fraction of the carriers, realizing an electrolyte-mediated chemo-electric process that enables diverse chemical-related synaptic functionalities. Based on this principle, the device achieves collaborative perception and integration of optical-chemical dual-modal signals through GaN nanowires. Zhou et al. demonstrated a novel multimodal resistive random access memory device array based on modified silk fibroin protein. This device array operates in two distinct modes: an optoelectronic RRAM mode characterized by unique negative–positive photoconductance memory, and an electrical RRAM mode featuring analog resistive switching capabilities (Fig. 9i) [10]. Hydroxyl bonds and carbon–oxygen double bonds in amino acid sequences provide active reaction sites for hydrogen bonding or polymerization, forming a series of traps that facilitate resistive switching behavior. These structural features make the modified silk fibroin protein highly suitable for constructing synapses capable of simultaneously sensing optical and electrical signals. Leveraging this mechanism, the device achieves collaborative perception and integration of optical-electrical dual-modal signals through the modified silk fibroin protein.

The defining characteristic of memristors lies in their dynamic resistive memory effect, where the conductance value evolves with the integral or pulsed characteristics of input signals, analogous to the plasticity of biological synapses [323]. This property enables memristors to inherently record the spatiotemporal correlations of multimodal inputs through their conductance states (or resistance values), providing a physical foundation for multimodal signal fusion [324]. Single-memristor multimodal sensing systems typically adopt two architectures. The first involves designing functional layer configurations with multilayer memristor stacks, each layer dedicated to specific signal modalities. A common functional layer is the ML layer, which quantitatively converts mechanical stimuli into light emissions in real-time and in situ. The total input voltage is proportionally distributed across layers, with conductance change rates determined by signal intensity. The second approach employs multifield-sensitive memristive materials (e.g., oxide and organic composites) whose resistance (conductance) simultaneously responds to multiple physical quantities (temperature, pressure, light, chemicals, etc.) [40, 325]. A prevalent signal fusion method is adaptive fusion based on memristive dynamics, where conductance evolution equations are established to integrate synergistic effects of multiphysical parameters [14, 326]. By adjusting pulse timing and width, the contribution weights of each modality to conductance are controlled. Another common strategy is dynamic encoding, categorized into time–amplitude hybrid modulation and frequency division multiplexing. Time–amplitude hybrid modulation maps low-frequency parameters (e.g., temperature) to steady-state conductance changes through direct current (DC) bias or slow-varying voltage applications [327, 328]. Simultaneously, high-frequency parameters (e.g., vibration) are encoded by superimposing alternating current (AC) excitation signals, utilizing memristive dynamic responses such as threshold switching to capture transient information [329]. In frequency division multiplexing, distinct excitation frequencies are assigned to each physical quantity (e.g., 1 Hz for temperature, 10 Hz for pressure, 100 Hz for light), with frequency-domain analysis (e.g., Fourier transform) decomposing modal contributions to conductance. The output conductance can be fed back to the input terminal, enabling unsupervised learning and online optimization by dynamically adjusting excitation amplitude-frequency parameters based on target response comparisons.

### Single-Sensor and Single-Memristor Multimodal Sensing System

Single-memristor multimodal sensing systems possess significant advantages of extremely simplified hardware architecture and exceptionally high integration density. This capability allows them to directly map multiple external stimuli onto unified resistive state changes to achieve hardware-level parallel information fusion, yet their core challenge lies in the concurrent alteration of the memristor's resistive state by multiple signals [330]. This makes it difficult for the system to effectively distinguish whether resistance changes originate from target signals (e.g., pressure) or environmental interference (e.g., temperature drift), preventing reliable separation of valid information [163]. Furthermore, the sensing capability of single memristors is constrained by their inherent physical properties, typically only permitting limited mode switching through external conditions (such as voltage or frequency adjustments), making it difficult to efficiently and simultaneously process multiple physical quantities [331]. More critically, as all signals share the same resistive change channel, the system is compelled to rely on complex algorithms (e.g., deep learning) for reverse analysis of mixed signals, increasing computational burden [332]. In contrast, the single-sensor and single-memristor multimodal sensing system employs dedicated sensors specifically designed for target physical or chemical quantities, enabling selective response to target signals. The sensor output exhibits strong correlation with target signals, significantly suppressing environmental interference. In this system, the sensor is responsible for detecting specific physical quantities (e.g., pressure), while the memristor senses another physical quantity and generates pulse outputs. Through collaborative operation, they fuse multimodal signals into a single pulse sequence, achieving data compression and efficient transmission. Simultaneously, this system supports collaborative customized design of sensors and memristors. For example, by adjusting parameters such as piezoresistive coefficient of the sensor, its response performance to specific target signals can be optimized [333].

**Fig. 10:**
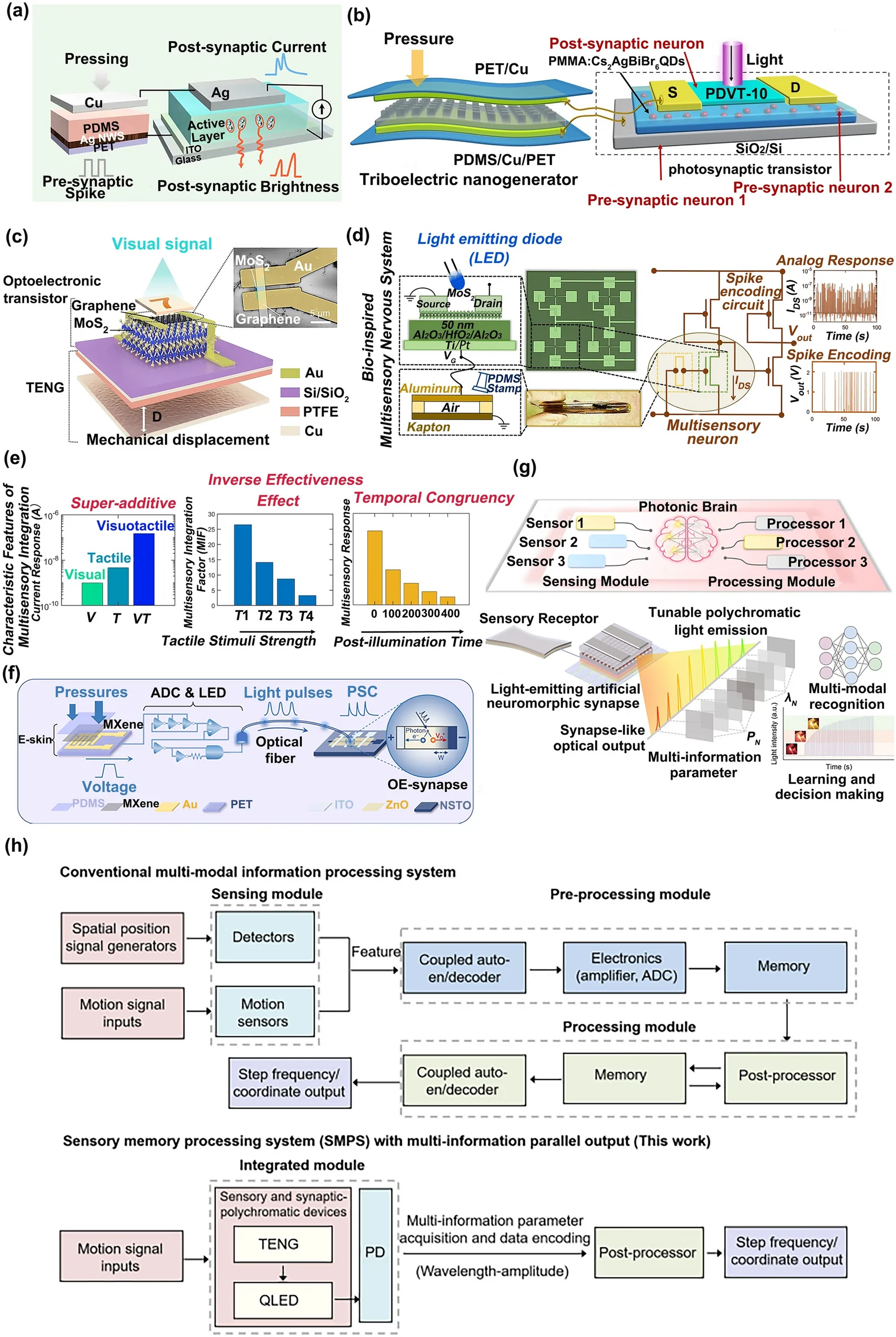
Single-sensor-memristor integration for multimodal sensing. **a** Photoelectric dual-output tactile sensing system. Reproduced with permission [334]. Copyright 2022, Nano Letters. **b** TENG-synaptic transistor multimodal nerve device. Reproduced with permission [335]. Copyright 2021, Nano Energy. **c** Optoelectronic-TENG artificial synapse. Reproduced with permission [336]. Copyright 2021, Science Advances. **d** Bio-inspired visuo-tactile neuron integrating triboelectric tactile sensor and monolayer MoS_2_ photo-memristor. Reproduced with permission [294]. Copyright 2023, Nature Communications. **e** Multisensory integration features of **d**. **f** Artificial afferent nerve systems integrating pressure sensor, ADC-LED circuit, and memristor. Reproduced with permission [338]. Copyright 2020, Nature Communications. **g** Photonic neuromorphic sensory memory system. Reproduced with permission [278]. Copyright 2023, Nature Communications. **h** Conventional versus parallel-output multimodal processing systems

In a multimodal sensing system composed of a single sensor and a single memristor, the sensor is typically a pressure sensor used to detect pressure signals, while the memristor serves triple roles in signal perception, fusion, and storage. Shan et al. reported a novel artificial tactile sensing system capable of sensing pressure and electrical signals simultaneously, and achieving parallel output of photonics and electronic signals (Fig. 10a) [334]. One end of the TENG is connected to the Ag terminal of the memristor, and the other end to the ITO terminal of the memristor. The TENG receives stimuli, converts them into action potentials, and transmits the generated signals to the memristor. This process triggers the memristor to produce electroluminescence and synaptic memory current signals. By coupling a lead-free perovskite-based synaptic transistor with a TENG, wu et al. proposed an artificial multimodal integration neuron capable of sensing pressure and optical signals (Fig. 10b) [335]. The two terminals of the TENG are connected to the source and gate of the floating-gate transistor, respectively. The flexible TENG serves as a skin receptor to convert external pressure signals into electrical signals, while the perovskite quantum dots in the floating-gate transistor act as retinal receptors to transform optical stimuli into electrical signals. Subsequently, the electrical signals converted by the TENG are transmitted to the gate of the floating-gate transistor (functioning as presynaptic neuron 1), and the electrical signals converted by perovskite quantum dots are captured by the floating gate (functioning as presynaptic neuron 2). These two presynaptic signals are integrated and converted into channel current at the source-drain terminal (acting as a postsynaptic neuron), mimicking biological EPSC. Yu et al. presented a bionic mechano-photonic artificial synapse with synergistic pressure and optical signals perception capabilities (Fig. 10c) [336]. The synaptic device is constructed from a graphene/MoS_2_ heterostructure-based phototransistor and an integrated TENG in contact-separation mode. The integrated TENG component comprises Cu/polytetrafluoroethylene (PTFE)/Cu conFig.d in a contact-separation mode. One triboelectric layer (PTFE/Cu) is connected to the transistor gate, while the opposing Cu electrode serves as a movable counter triboelectric layer. Mechanical displacement between dual tribolayers of the TENG induces triboelectric potential coupling into the transistor. This coupling mechanism directly governs charge transfer/exchange in the graphene/MoS_2_ heterostructure channel by Fermi level of modulating graphene and energy band alignment of MoS_2_, thereby enabling photonic synaptic current modulation. The device achieves photonic synaptic plasticity through the combined action of mechanical displacement (acting as a state parameter) and light pulses that reflect spatiotemporal information (e.g., intensity and illumination time).

In the aforementioned multimodal sensing systems composed of a single sensor and a single memristor, the pressure sensor is directly connected to the gate terminal or both ends of the memristor, transmitting voltage signals to the memristor for further fusion. While this approach simplifies the hardware architecture, it risks constraining the signal dynamic range. The output signals from the pressure sensor may exceed the operational range of the memristor, leading to signal saturation or nonlinear distortion. Additionally, such systems may exhibit compromised noise immunity and restricted cross-modal synergy capabilities. Sadaf et al. introduced a bio-inspired visuo-tactile multisensory neuron comprising a triboelectric tactile sensor, a monolayer MoS_2_ photo-memristor and an associated spike encoding circuit (Fig. 10d) [294]. The bionic visuo-tactile multisensory neuron integrates a tactile sensor connected to the gate terminal of a monolayer MoS_2_-based photonic synaptic transistor and associated spike encoding circuitry. The tactile sensor employs triboelectric effects to transduce pressure stimuli into electrical spikes, which are subsequently mapped to source-drain output current spikes through channel conductance modulation. Meanwhile, optical stimuli are encoded as threshold voltage shifts via the photogating effect in the monolayer MoS_2_ synaptic transistor, enabling light-driven channel conductance regulation. Through synergistic modulation of channel current by both optical and pressure signals, bio-inspired neuromorphic integration of light-force-electrical signaling is achieved. The bionic visuo-tactile multisensory neuron demonstrates three characteristic features of multisensory integration: super-additive responses to cross-modal cues, inverse effective effect, and temporal congruency (Fig. 10e). Super-additive response refers to the phenomenon where the neural response intensity elicited by cross-modal combined stimuli significantly exceeds the algebraic sum of unimodal responses. In the multisensory neuron, the reaction induced by pressure-optical signal integration surpasses that obtained through single-modality integration. Inverse effective effect describes the enhanced multisensory integration when unimodal signals are weak. The physical origin of this effect in the multisensory neuron lies in the screening of triboelectric gate voltage (generated by tactile stimuli) through trapped charges at the interface induced by visual stimulation. Temporal congruency requires temporal synchronization of cross-modal signals. The physical origin of temporal congruency can be attributed to the fact that the persistent photocurrent in the photonic synaptic transistor directly results from photo-induced carrier trapping at the MoS_2_/dielectric interface. De-trapping dynamics gradually restore the device to its pre-illumination conductance state over time.

Unlike conventional multimodal systems that directly convert pressure signals detected by the sensor into voltage inputs for the memristor, another prevalent strategy involves inserting an ADC between the pressure sensor and memristor to transform pressure information into optical pulses [337]. Tan et al. developed an optoelectronic spiking afferent nerve system composed of an ITO/ZnO/Nb:SrTiO_3_-based synaptic optoelectronic memristor and an MXene-based pressure sensor. This system demonstrates neuro-encoding, perceptual learning, and memory capabilities to emulate pressure and optical signals sensing and processing (Fig. 10f) [338]. The system senses pressure through the MXene-based sensor, converts pressure information into optical pulses by coupling light-emitting diodes to ADC circuitry, and subsequently integrates these optical pulses using the synaptic optoelectronic memristor. Importantly, the synaptic weight changes of optoelectronic memristor at run-time by the input pressure signal because of a photomemristive effect, and the weight change depends on the pressure amplitude.

The aforementioned multimodal neuromorphic systems rely on discrete components, and their hardware integration remains constrained. Shan developed an efficient sensory memory processing system capable of processing sensory information while generating synaptic-like multichromatic light outputs, enabling diversified optical utilization in information processing and multimodal recognition (Fig. 10g) [278]. This system employs a TENG as sensory receptors and QLED devices as luminescent neuromorphic synapses. The sensory memory processing system achieves synaptic-mimetic multi-wavelength optical signaling through synaptic multicolor emission, facilitating multimodal information recognition via artificial neural networks. The TENG serves as sensory receptors that collect tactile signals through contact-separation motions with skin, converting them into presynaptic voltage pulses. These electrical signals drive the artificial synaptic devices to simultaneously generate electroluminescence and modulate postsynaptic currents. The hybrid quantum dots in the emissive layer enable electric-field-tunable color emission, where spectral output can be dynamically tuned by adjusting the applied electric field intensity. Through this mechanism, the sensory memory processing system realizes synaptic-adaptable multiband optical outputs by regulating contact-induced electric field strength. Unlike conventional multimodal systems requiring separate sensory modules, isolated memory processors, and complex encoder-decoder couplings, this integrated sensory memory processing system with parallel multi-information outputs (Fig. 10h). The design significantly reduces circuit complexity while maintaining efficient sensory signal processing capabilities.

The above discussion outlines several common device architectures and multimodal fusion methods for single-sensor and single-memristor multimodal sensing systems. In systems composed of a single sensor and a single memristor, the sensor typically employs a TENG to detect pressure signals, while the memristor concurrently serves three functions: signal sensing, fusion, and storage [339]. A conventional architecture directly connects the TENG to the memristor terminals or gate. The TENG transduces mechanical stimuli into action potentials and transmits the generated signals to the memristor. These signals directly modulate the conductance state of memristor, synergistically interacting with potentials derived from the intrinsic sensing capabilities of memristor (e.g., light or temperature responses) to achieve multimodal signal coupling [340]. The primary fusion strategy involves dynamic weight adaptive fusion, where algorithms adjust the weighting between pressure and memristor-derived signals in real time. An alternative architecture routes sensed pressure signals through dedicated processing modules (e.g., spike encoders or ADCs) to convert analog pressure data into electrical spikes or optical pulses [341]. These transformed signals may manifest as voltage pulses with modulated amplitudes and frequencies, or optical pulses with tunable frequencies and widths [342]. Subsequently, the processed signals are transmitted to the memristor to dynamically regulate synaptic weights. In such architectures, pulse-coding-based spatiotemporal fusion predominates. Analog pressure signals are encoded into pulse sequences (e.g., pulse frequency modulation), which are then fused with the pulse responses of memristor (e.g., pulse width modulation) triggered by light or temperature via synaptic plasticity rules like STDP [163]. The temporal correlation between pressure-induced pulses and memristor-generated pulses dynamically adjusts the conductance weights, enabling adaptive multimodal integration [316].

### Multi-Sensor and Single-Memristor Multimodal Sensing System

Although single-sensor and single-memristor multimodal sensing systems can selectively respond to target signals and effectively suppress environmental interference, their core challenge lies in the fact that the single-sensor design typically targets specific physical quantities (for example, using piezoelectric materials for pressure detection) [343]. The inherent linear response characteristics constrain their applicability across the amplitude ranges of multimodal signals. When a single sensor responds to multiple physical quantities, signals are prone to mixing during the conversion process, and compromise design is required among the response characteristics of various physical quantities [344]. In contrast, within multi-sensor and single-memristor multimodal systems, each sensor can be independently customized for target signals, expanding the sensing dimension through the collaborative operation of chemical/biosensors and physical sensors. Different sensors independently detect specific physical quantities, fundamentally eliminating signal crosstalk [345]. When a single sensor fails, the remaining sensors can still continuously provide partial modal data. Each sensor can independently set its measurement range to avoid signal saturation, while the memristor time-sequentially processes signals from different sensors through a switching mechanism, effectively reducing instantaneous load [238].

**Fig. 11:**
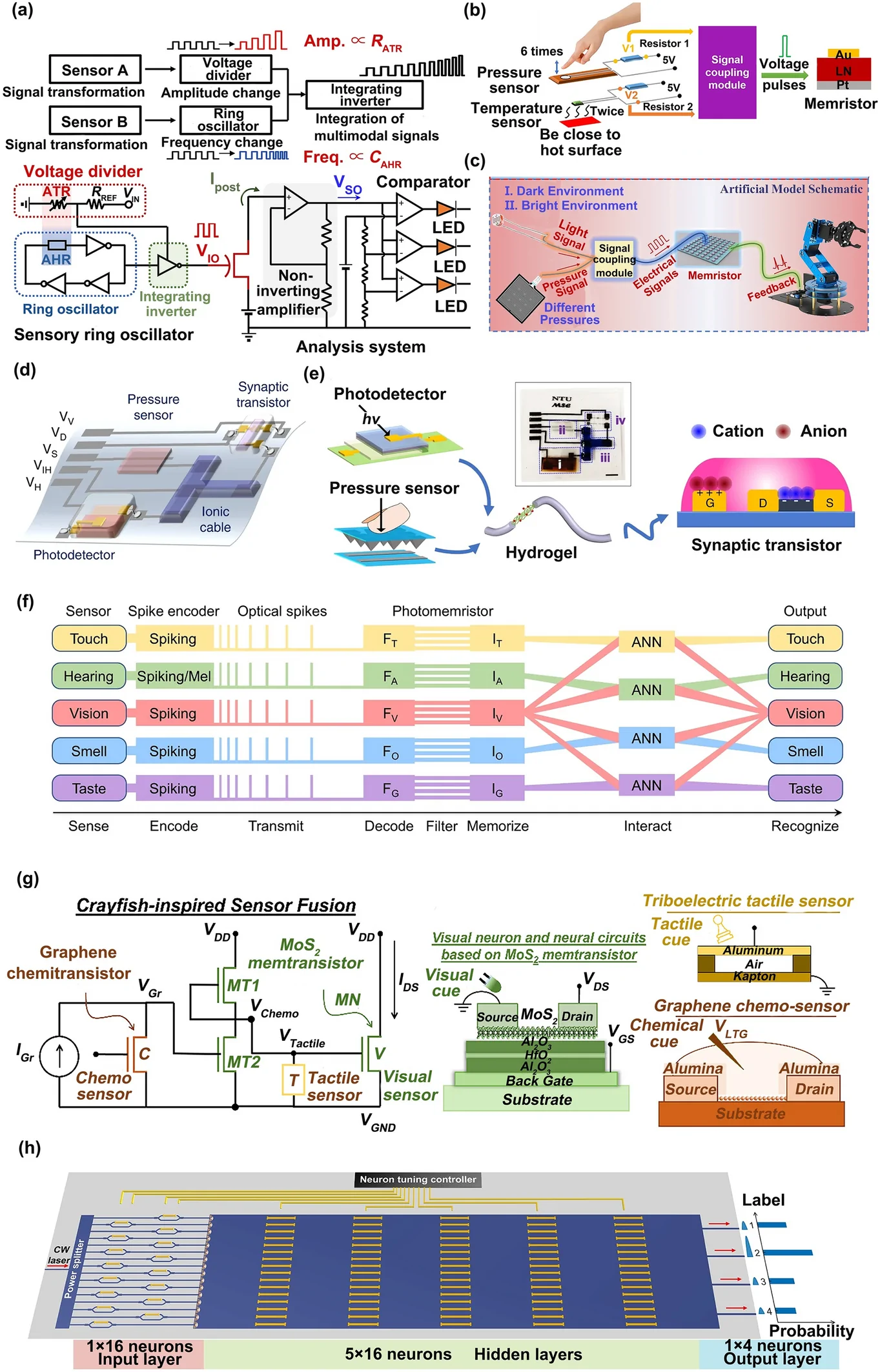
Memristor-multisensors integration for multimodal sensing. **a** Sensory ring oscillator circuit and multimodal integration system. Reproduced with permission [346]. Copyright 2021, ACS Materials Letters. **b** Bio-inspired sensory memory system with pressure/temperature sensors. Reproduced with permission [347]. Copyright 2022, Advanced Intelligent Systems. **c** Multimodal sensory memory system with photosensitive/pressure sensors and robotic arm. Reproduced with permission [348]. Copyright 2023, InfoMat. **d** Bimodal artificial sensory neuron for visual-haptic fusion. Reproduced with permission [26]. Copyright 2020, Nature Communications. **e** Bimodal artificial sensory neuron patch integrating photodetector, pressure sensor, hydrogel and memristor. **f** Multisensory neural network operation. Reproduced with permission [20]. Copyright 2021, Nature Communications. **g** Crayfish-inspired sensor fusion architecture integrating photosensitive memtransistors, triboelectric tactile sensor, and graphene-based chemitransistor. Reproduced with permission [341]. Copyright 2024, Nano Letters. **h** Multimodal classification optical neural network. Reproduced with permission [350]. Copyright 2024, Nature Communications

In a multimodal sensory system based on multiple sensors and a single memristor, the fusion of multimodal signals typically requires additional circuit components to convert the voltage signals acquired from different sensors into other signal forms. Kim et al. presented an artificial multimodal integration system capable of simulating discomfort perception based on the integration of multiple sensory signals (Fig. 11a) [346]. The system consists of MXene-based artificial sensors, a ring oscillator, and an EDL synaptic transistor. The artificial temperature receptor and humidity receptor in the system detect ambient temperature and humidity, respectively, converting them into electrical signals. These signals are then transmitted to the sensory ring oscillator. Within the sensory ring oscillator, each sensor converts external stimuli into resistive and capacitive signals, respectively. The integrating inverter subsequently translates changes in resistive and capacitive signals into voltage pulse amplitude and frequency. The integrated voltage pulses are applied to the synaptic transistor, which converts them into postsynaptic currents.

Unlike ring oscillators that convert sensed signals into voltage pulse amplitudes and frequencies, another multimodal sensing system adopts a distinct signal fusion method by transforming induced voltages into corresponding pulse sequences through a signal coupling module. Pan et al. proposed a novel universal signal-coupling method for applying stimuli from different sensors to the memristor (Fig. 11b) [347]. Based on the proposed signal coupling method and fabricated memristors, an artificial sensory-memory system incorporating sensors (pressure sensors and temperature sensors) and memristors has been realized. A method termed “indirect signal coupling” has been proposed as a universal signal coupling approach. This method requires setting a threshold voltage. When voltage signals reach the threshold voltage, fixed voltage pulses are applied to the memristor to alter its conductance. Flexible pressure sensors and temperature sensors are connected in series with resistors. Once their respective voltage responses exceed the threshold voltage, fixed voltage pulses are applied to the memristor, thereby modifying its conductance. After applying each fixed pulse sequence, the resistance of memristor gradually decreases. The conductance of memristor varies with the quantity of external stimuli received by the sensors. Consequently, the altered memristor resistance reflects cumulative effects of multiple past external stimuli received by the sensors. However, this multimodal signal integration system requires multiple redundant resistors, leading to high hardware complexity. Yan et al. have proposed an improved design to address this issue. Yan et al. proposed an ultra-stable artificial multisensory sensory memory system with visual and tactile functions by combining a pressure sensor, a photosensitive sensor, a signal coupling module, a synaptic device, and a robotic arm (Fig. 11c) [348]. The sensing-memory system collects optical and pressure information from photosensitive and pressure sensors, respectively. Signals generated by both sensors are input to a signal coupling module, which calculates and processes the signals. The generated corresponding pulse sequences are then sent to the memristor, enabling observation of memristor current signal changes that effectively identify environmental parameters (pressure and light intensity) where the system resides. The proposed signal coupling method also requires setting a threshold voltage. When voltage signals reach this threshold, fixed-sequence voltage pulses are applied to the memristor.

The aforementioned approaches utilizing signal coupling modules or ring oscillators to process voltage signals sensed from sensors require complex circuitry, resulting in high hardware complexity, along with increased cost and power consumption. Wang et al. have addressed this issue by employing ion-conductive cables and hydrogels to replace traditional coupling modules. Wang et al. developed a bimodal artificial sensory neuron to implement the visual-haptic sensory fusion processes (Fig. 11d) [26]. The bimodal artificial sensory neuron consists of four core components: a resistive pressure sensor, a perovskite-based photodetector, a hydrogel-based ionic cable, and a synaptic transistor (Fig. 11e). The bimodal artificial sensory neuron collects optical and pressure information from the photodetector and pressure sensor, respectively, transmits the bimodal information through the ionic cable, and integrates them into postsynaptic currents via the synaptic transistor. The resistance of photodetectors and pressure sensors decreases with increasing incident light intensity or applied pressure. As the sensor resistance drops, ionic flux is induced through ionic cables, with the fluxes from both sensors converging within the hydrogel matrix. Since the opposite side of the hydrogel is connected to the gate of a synaptic transistor, the accumulated ions electrostatically couple to the EPSC through the semiconductor channel of the transistor. The fusion of pressure and optical signals is achieved through integration effect of the synaptic transistor on multiple inputs, which can be mathematically described as the integral of the product of input intensity and its distance-dependent weight.

The aforementioned signal fusion methods typically rely on voltage signals or converting voltages into fixed pulse sequences. Another common multimodal signal fusion approach involves voltage spike encoders that encode electrical potentials into optical spikes for communication. This method effectively mitigates voltage attenuation and parasitic resistance issues during sensor data transmission. Tan et al. reported a bio-inspired spiking multisensory neural network that integrates artificial touch, hearing, vision, and simulated smell and taste with cross-modal learning via artificial neural networks (Fig. 11f) [20]. With distributed multi-sensor arrays and biomimetic hierarchical architectures, the spiking multisensory neural network can not only perceive, process, and memorize multimodal information but also fuse multisensory data at both hardware and software levels. The system senses multimodal physical stimuli through various detectors and converts them into voltage signals. Spike encoders encode potentials into optical spikes for communication. Photonic memristors integrate optical spikes, decode multisensory information, filter and memorize environmental data. Finally, artificial neural networks combine cross-modal signals with associative learning. Sensory inputs dynamically alter spike rate and postsynaptic currents of the photonic memristor during operation via persistent photoconductive effects, enabling built-in memory of sensory information. The inherent memory and information filtering properties of photonic memristor array facilitate supervised training of artificial neural networks, establishing associations across five sensory modalities to achieve advanced cognitive capabilities.

Conventional data fusion strategies typically involve collecting information from individual sensors and transmitting it to a signal fusion module, where signal formats are further transformed. This approach lacks a critical aspect known as cross-sensor modulation, where one or more sensors directly modulate responses of each other. Furthermore, this strategy overlooks the intrinsic synergies and dependencies between sensor modalities. Sakib et al. proposed a neuromorphic platform integrating graphene-based chemical transistors, monolayer MoS_2_-based photosensitive memtransistors, and triboelectric tactile sensors to enable the cross-modal integration of chemical, optical, and pressure signals (Fig. 11g) [341]. In the neuromorphic platform, the tactile sensor is directly connected to the gate terminal of the memtransistor. In contrast, the output of the graphene chemical transistor is first amplified using a MoS_2_-based thin film transistor inverter amplifier before being fed to the gate terminal of the memtransistor. Finally, by leveraging the light-controlled effect observed in the memtransistor, optical signal is encoded as threshold voltage shifts. Since the TENG is connected to the gate terminal of the memtransistor, electrical pulses generated by touch are encoded as current spikes at the output of memtransistor. As the channel conductance can be controlled by applying an electrical bias to the chemical solution, electrical pulses generated by chemical signal serves as the gate bias for the memtransistor, enabling tactile responses to be modulated by chemical signals. The light-controlled effect observed in the memtransistor encodes optical signal as threshold voltage shifts, thereby achieving modulation of tactile responses. Stronger visual and chemical signals lead to enhanced responses due to the combination of a more negative threshold voltage and a more positive read voltage. Several distinct signals enable synergistic modulation, where the integrated effect of multiple sensory signals not only exceeds individual responses to each signal but also surpasses their linear summation.

All the aforementioned multimodal neuromorphic systems rely on discrete components, and their hardware integration remains constrained [349]. Cheng et al. reported a trainable diffractive optical neural network architecture to process and classify multimodal data by light propagation (Fig. 11h) [350]. By leveraging superposition and coherence properties of optical signal, large-scale neurons in hidden layers can be naturally connected through diffraction under multimodal configurations. The trainable diffractive optical neural network comprises an input layer, five hidden layers, and an output layer. After feature extraction and fusion, a feature vector derived from multimodal datasets of different modalities such as vision, audio and pressure, which serves as the neural network input. The dimension of feature vector matches the number of neurons in the input layer, with each vector element encoded into optical signals via intensity modulation. In hidden layers, neurons are arranged in multilayer layouts, where connection weights between neurons are adjusted during training to achieve target functionalities.

The aforementioned discussion outlines several common multimodal fusion methods for multi-sensor and single-memristor multimodal sensing systems. Typically, multiple sensors first perceive distinct signals and convert them into voltage signals. Conventional multimodal fusion approaches typically involve transmitting sensed voltage signals to specialized processing modules (e.g., signal coupling modules, ring oscillators, or spike encoders) for converting the acquired electrical signals [346–348]. These may include fixed-frequency pulse trains, voltage pulses with modulated amplitudes and frequencies, or optical spikes with tunable frequencies and widths. The transformed signals are then delivered to memristors to dynamically adjust synaptic weights. In order to solve the impedance matching problem between memristors and different types of sensors, scaling resistors can be introduced to adjust the operating resistance of various sensors to the resistance range compatible with memristors [351]. Notably, ionic cables and hydrogels can substitute conventional processing modules, replacing voltage-based signaling with ionic flux variations to enable signal transmission and fusion [26]. However, conventional multimodal fusion methods lack cross-sensor modulation, where multiple sensors directly modulate responses of each other, thereby neglecting the intrinsic synergies and interdependencies between sensory modalities [352]. To achieve cross-sensor modulation, memristor-mediated sensor interconnections can be implemented. By interconnecting the outputs of different sensors through a memristor crossbar array, the signal transmission strength is dynamically regulated via the conductance states of memristor [332]. Encoding sensor signals into distinct pulse sequences (e.g., pulse frequency or phase modulation) enables cross-modal weight adaptation through the STDP rule at memristive synapses [353]. Additionally, the electrical pulses converted from one signal modulate the memristor channel conductivity, which acts as a gate bias to influence the electrical pulses derived from another signal. This mechanism establishes intrinsic synergies and interdependencies among multimodal signals.

### Summary and Challenges

In the development of multimodal sensing and fusion technologies, diverse architectural designs exhibit distinctive performance characteristics and application potentials due to differences in core principles, hardware support, and scenario adaptability. This section systematically compares the advantages and limitations of three representative multimodal architectures, providing references for subsequent technical refinement and scenario-specific selection. The multi-sensor and single-memristor neuromorphic architecture employs discrete high-performance sensors (e.g., optical, pressure, and chemical) to capture modality-specific signals. Following preprocessing, these signals are fed into a shared memristor array for fusion computation. This design maintains compatibility with existing sensing technologies while enabling high-precision, wide-bandwidth signal acquisition, with memristors processing only pre-encoded signals to reduce design complexity [308]. However, physical isolation between sensors introduces spatial mismatch requiring complex cross-modal calibration, inevitably creating hardware redundancy and necessitating sophisticated synchronization algorithms for data fusion [354]. Its fusion mechanism implements feature-level integration through memristive weighted fusion after analog-to-digital or pulse encoding of sensor signals, making it particularly suitable for applications demanding stringent single-modality accuracy such as biomedical multi-parameter monitoring [348]. In contrast, the single-sensor and single-memristor architecture significantly reduces hardware redundancy, eliminates synchronization challenges, and enhances noise immunity and stability [355]. Nevertheless, it requires sensors with cross-modal response capabilities, posing significant material design challenges. Concurrently, signal coupling introduces crosstalk and constrains the dynamic range within individual sensing units. Its primary fusion strategy employs dynamic adaptive weighting that continuously adjusts weights between sensor-derived and memristor-processed signals [356]. This configuration is ideal for space-constrained edge intelligence devices like wearable health monitors. Conversely, the single-memristor architecture integrates sensing and computation within homogeneous memristive elements, achieving minimal hardware complexity without signal conversion losses, thereby enabling low-latency operations and ultralow power consumption [357]. Challenges include co-optimizing multimodal sensitivity with accuracy, limited signal dynamic range, and significant fabrication complexities [358]. Its fusion mechanism leverages stimulus-specific energy thresholds or temporal scales to differentially drive resistance state transitions, accomplishing feature extraction and fusion directly at the device level [331]. The single-memristor approach excels in high-efficiency real-time processing scenarios such as neuromorphic vision for dynamic environmental perception [305].

Multimodal neuromorphic systems still face significant challenges in data conversion and fusion. A critical issue lies in the requirement for additional conversion modules to achieve spike encoding of multimodal signals. To address this limitation, the nonlinear threshold switching characteristics of memristors can be leveraged to directly map multimodal analog signals into spatiotemporal pulse sequences [359]. This can be implemented through cross-modal threshold modulation by designing differentiated voltage thresholds for distinct physical quantities, where composite input signals exceeding these thresholds spontaneously trigger conductance transitions and spike generation in memristors [40]. Alternatively, pulse frequency-intensity correlation enables signal strength modulation through input amplitude or duty cycle [327]. High-amplitude signals generate high-frequency spikes while low-amplitude signals produce low-frequency pulses, thereby eliminating the need for external frequency modulation circuits. The most efficient approach exploits multi-physical field coupling effects to achieve concurrent multimodal perception and fusion within single memristor-based multimodal systems [360]. Another fundamental challenge involves potential information loss during multimodal data fusion, particularly the suppression of weak signal features. This issue can be mitigated through coordinated hierarchical feature preservation strategies and adaptive fusion mechanisms [361]. During the preprocessing stage, dedicated independent units within the memristor array implement dual-channel parallel processing. One channel preserves raw signals through lossless or low-compression encoding techniques such as pulse interval modulation or time–amplitude hybrid coding, enabling direct storage or transmission of original data streams [362]. The complementary channel extracts multimodal joint features through spatiotemporal filtering or sparse coding algorithms. This architecture effectively balances the requirements for information fidelity and fusion efficiency while maintaining hardware compactness, providing a hardware–software co-design paradigm for robust neuromorphic perception systems.

## Conclusion and Perspective

In summary, with the advancement of the Internet of Things era, the application scenarios and modality recognition requirements of neuromorphic devices/systems have become increasingly diversified, driving growing research enthusiasm for multimodal/multi-task recognition. Herein, we investigate the complex physical mechanisms underlying multimodal neuromorphic devices, focusing on six distinct resistive switching mechanisms: charge trapping, ion migration, electrochemical doping, conductive filament formation, ferroelectric polarization, and phase transition. The working principles of these mechanisms in sensing various input signals are systematically elucidated. A comprehensive analysis is presented regarding their implementation strategies for multimodal perception. This analysis reveals that electrochemical doping and ion migration mechanisms demonstrate superior applicability in multimodal signal fusion due to their exceptional linearity, wide dynamic range, and direct signal transduction capabilities. Furthermore, the study categorizes multisensory neuromorphic devices into three architectural classifications. It then examines diverse multisensory fusion approaches and signal processing techniques within each category, aiming to effectively process multisensory stimuli and construct high-efficiency neuromorphic sensory systems. Finally, the current challenges in multimodal perception systems are critically summarized, accompanied by forward-looking perspectives on their future development directions.

Multimodal neuromorphic perception systems still confront multiple challenges in multimodal fusion. The limited modality scalability of neuromorphic hardware, constrained by synaptic precision, restricts existing memristor arrays from effectively distinguishing subtle cross-modal differences due to insufficient conductance modulation accuracy [142, 363]. Simultaneously, SNNs rely on discrete spike-based encoding, which exhibits inferior ability to capture continuous features of high-frequency vibration signals (> 1 kHz) compared to conventional ADC sampling [364]. Information loss and weak signal suppression persist in neuromorphic devices, particularly when multimodal signals share neural buses, where pulse collision-induced information degradation occurs [163, 363]. High-energy modalities (e.g., intense illumination) tend to overwhelm low-energy signals (e.g., infrared thermal radiation), while analog-to-pulse conversion inevitably sacrifices fine-grained signal characteristics [365]. Furthermore, dynamic environmental adaptability remains problematic as data quality fluctuates with contextual variations, necessitating real-time adjustment of multimodal data weights and fusion strategies. Current static-environment-oriented algorithms, such as attention mechanisms and weighted fusion, demonstrate limited effectiveness against abrupt interference. Another critical limitation arises from the inherent heterogeneity across modalities. Disparities in sampling rates and spatiotemporal resolution between different sensory signals create alignment challenges, necessitating sophisticated dynamic coordination mechanisms to resolve information redundancy and conflicts [289, 366]. The disparities in sampling rates across sensory signals originate from the inherent timescales of their physical processes: visual sensors typically operate at kilohertz frequencies, while temperature or gas sensors require millisecond-to-second response times [367, 368]. Spatial resolution also varies with sensor size and pixel density, causing divergent spatial granularity for the same event across the array [369]. During real-time fusion within a unified processing window, fast signals may recur multiple times before slower signals update, inducing temporal misalignment. Consequently, rapidly evolving visual cues (e.g., object motion) fail to synchronize with slower thermal responses, creating inconsistent feature representations [370]. Simultaneously, high-resolution channels may spatially oversample low-resolution regions, triggering spatial mismatch [371]. These discrepancies intensify in dynamic environments. For example, when visual and tactile sampling rates differ by an order of magnitude, fusion accuracy degrades sharply with increased error [372]. Addressing this demands programmable delay lines, interpolation-based alignment, and dynamic weighting mechanisms at either device or algorithmic levels to achieve spatiotemporal synchronization [373]. These challenges collectively underscore the imperative for hardware-algorithm co-optimization to advance neuromorphic multimodal integration.

The future development of multimodal neuromorphic perception systems in multimodal fusion will revolve around hardware innovation, algorithmic optimization, and revolutionary application scenarios. Advancements will focus on breakthroughs in bio-inspired hardware and heterogeneous integration technologies, enabling deeper emulation of biological parallel processing mechanisms through the intrinsic fusion of multimodal sensors and neuromorphic devices. This progression will extend to broader modality integration while achieving precision improvements in cross-modal discrimination. Next-generation systems will implement hybrid pulse-analog encoding strategies and dynamic decoupling mechanisms to address high-frequency signal acquisition and information preservation challenges, synergistically combining the efficiency of pulse-based encoding with the continuity of analog signal representation [374]. To enhance adaptability in dynamic environments, these systems will incorporate reinforcement learning-driven weight adjustment mechanisms that dynamically optimize modality confidence levels based on real-time environmental changes. To reduce power consumption and latency, future systems must promote compute-in-memory architectures and edge computing networks, enabling edge computing and energy efficiency optimization [375]. The application landscape will undergo transformative expansion, including real-time collaborative perception through edge-deployed multimodal devices (e.g., smart speakers with cameras and sensors) for intelligent IoT ecosystems, neuroprosthetics decoding electromyography and electroencephalography signals via neuromorphic processing, and immersive virtual-physical interaction systems integrating visual, auditory, and tactile feedback. With the maturation of optoelectrochemical multifield-coupled devices, communication-perception co-design frameworks, and context-aware adaptive algorithms, such systems are poised to achieve unprecedented environmental adaptability and energy efficiency. These gains will be realized across applications in robotics, autonomous vehicles, and medical electronics, ultimately driving intelligent upgrades in the closed-loop perception-decision-action paradigm.
